# Tolerance and mycoremediation of silver ions by *Fusarium solani*

**DOI:** 10.1016/j.heliyon.2020.e03866

**Published:** 2020-05-12

**Authors:** Manal T. El Sayed, Ashraf S.A. El-Sayed

**Affiliations:** Botany and Microbiology Department, Faculty of Science, Zagazig University, Zagazig, 44519, Egypt

**Keywords:** Biotechnology, Microbiology, Plant biology, *Fusarium solani*, Ag(I), Stress, Antioxidant enzymes, Biosorption, Nanoparticles, Biological activity, Metabolite

## Abstract

Silver ions discharged from various industries, are potentially toxic to living organisms at low concentrations, thus, there is an increasing need for development of an eco-friendly and cost-effective approach for its bioremediation. Filamentous fungi especially, *Fusarium solani* displayed a strong resistance to copper and cadmium ions as revealed from our previous study (El-Sayed 2014), however, the mechanisms of silver resistance by this fungus has not been resolved yet. Thus, this study was an extension to our previous work, to elucidate the mechanism of silver ions resistance and biotransformation by *F. solani*. The growth, bioaccumulation, thiol, total antioxidant, malondialdehyde (MDA), hydrogen peroxide (H_2_O_2_) contents and polyphenol oxidase (PPO) and catalase (CAT) activities of *F. solani* in response to silver ions were determined. Production and bioaccumulation of silver nanoparticles was characterized by UV-visible spectroscopy, TEM, and X-ray powder diffraction (XRD). The ultrastructural changes of *F. solani* induced by Ag(I) was examined by TEM and SEM. Production of oxalic acid by *F. solani* was increased by about 343.8% in response to 400 mg/l Ag(I), compared to control cultures (without silver ions) as revealed from HPLC analysis. The maximum biosorption levels by the native and alkali-treated biomass were carried out at pH 5.0, initial metal concentration 200 mg/l, biomass 0.5 g/l, temperature 35 °C, and contact time 1 h (native biomass) and 3 h (alkali-treated biomass). Fourier transform infrared spectroscopy (FTIR) results revealed that the main functional groups involved on this mycoremediation were C–S stretching, C=O C=N, C – H bending, C–N stretching and N–H bending. EDX spectra indicated the involvement of fungal cellular sulfur and phosphorus compounds in Ag(I) binding.

## 1 Introduction

Toxic heavy metals are the main environmental pollutants due to their non-biodegradable nature, abundance, biomagnifications, and accumulation in the food chain (Mahmoud et al., 2015). Silver (Ag(I)) ions have been used frequently in various industries and medical applications, for its antimicrobial properties and higher electrical and thermal conductivity (Massarsky et al., 2014). Natural processes and anthropogenic activities account about 18 and 82 %, respectively, of the evaluated 2,430 tons of Ag(I) entering the environment every year (Yin et al., 2019). Exposure to dust containing relatively higher levels of Ag(I) ions may cause breathing problems, lung and throat irritation, and stomach pain (Agency for Toxic Substances and Disease Registry (ATSDR) 2011). Exposure of living cells to Ag(I) inactivates the biological functions of their nucleic acids and proteins by binding to the carboxyl, thiol, amino, imidazole, and phosphate groups (Rathnayake et al., 2010). Thus, removal of silver ions from wastewater is an environmental and public necessity.

Many technologies like ion exchange, chemical precipitation, and membrane filtration have developed to remove heavy metal ions from polluted areas. However, chemical precipitation usually creates low-density sludge and cause extra secondary pollution (Sao et al., 2017). The membrane filtration and ion exchange approaches are generally complicated and costly. Therefore, an alternative simple, eco-friendly and low-cost remediation method is urgently required. Bioremediation of the environmental pollutants by microorganisms or their metabolites has been widely reported. Among the microorganisms utilized for bioremediation are bacteria (Poornima et al., 2014; Jiang et al., 2015), fungi (Liu et al., 2017; Bano et al., 2018), and algae (Piccini et al., 2019). Metal-microbe interactions include bioleaching, bioaccumulation, biotransformation, biomineralization, and biosorption (Banerjee et al., 2018). The active and metabolically mediated uptake of heavy metals by living biomass (bioaccumulation) is different from the passive metal sequestering by dead biomass (biosorption) (Naja and Volesky, 2011). Passive diffusion, facilitated diffusion, and active transport are the three pathways of metal bioaccumulation in microbes (Banerjee et al., 2018). Many mechanisms like ion exchange, covalent bonding, and adsorption, can occur in living and dead biomass. Therefore, biosorption is a suitable alternative for the traditional methods for metal removal (Banerjee et al., 2018).

Microorganisms can resist stressful situations with toxic heavy metals. The study of microbial tolerance mechanisms is very crucial to find out efficient bioremediation approaches (Yin et al., 2019). The main tolerance pathways of heavy metal ions by microorganism involve (a) intracellular sequestration *via* sulfides, cytosolic polyphosphates and metallothionines, (b)extracellular sequestration *via* many biological structures, e.g. biosurfactants, siderophores, glutathione, and extracellular polymeric substances, (c) active transport by ABC transporters, proton-cation antiporters, P-type efflux ATPase, and (d) enzymatic detoxification (Iskandar et al., 2011; Salunkhe et al., 2011; Sabatini et al., 2016; Maizel et al., 2016; Cecchi et al., 2017; Vargas-García et al., 2012; Amirnia et al., 2015; Oves et al., 2017).

Bacteria can absorb heavy metal ions on the polysaccharide slimy layers and extracellular polymeric substances (EPS) via anionic and cationic functional groups. Nevertheless, isolation, screening, and separation of bacterial pellets on a larger-scale may be difficult but still considered as one of the effective ways for pollutant remediation (Fang et al., 2010; Yue et al., 2015). Fungi gained widespread interest because of the cost-effective handling and culturing on a large-scale, high tolerance and accumulation of elevated heavy metal concentrations, the easy separation of fungal biomass, and their ability to grow under extreme conditions of pH, temperature, and a shortage of nutrients (Salunkhe et al., 2011; Mustapha and Halimoon, 2015). Besides, fungi able to secrete extracellular enzymes for the assimilation of polysaccharides for former hydrolysis make capable the degradation of various pollutants. The glucuronic acid, chitin-chitosan complex, polysaccharides, and phosphate in fungal cells play a crucial role in biosorption (via coordination and ion exchange). Moreover, the cell surface of fungi is negatively charged due to the presence of ionizable sites and many functional groups like amine, hydroxyl, phosphate, carboxyl, and sulfhydryl groups that affect the biosorption capacity and specificity of fungal strains to metal ions (Maghsoodi et al., 2007).

The rate of biosorption was strongly affected by the nature of fungal biomass, chemistry of metal, and environmental factors (Siddiquee et al., 2015). A solute transferred from the bulk of solution to the liquid film around the biosorbent particles, and to the surface of particles (external diffusion), then diffused to bind to the active binding sites and interact with them (Alimohammadi et al., 2017). *Cochliobolus lunatus, Aspergillus fumigatus, Trichoderma harzianum* and *Amanita submembranacea* can accumulate Ag(I) and form Ag nanoparticles (AgNPs) (Salunkhe et al., 2011; Sabatini et al., 2016; Cecchi et al., 2017; El-Sayed et al., 2017; El-Sayed and Ali, 2020). Live, dead, pre-treated and immobilized forms of *Aspergillus* spp., *Penicillium* sp., *Botrytis* sp., *Trichoderma* sp., *Saprolegnia* sp., *Neurospora* sp., *Termitomyces clypeatus*, and *Saccharomyces cerevisiae*were applied frequently to remove toxic metal ions (Amirnia et al., 2015; Iram and Abrar, 2015; Mohammadian Fazli et al., 2015; Dhal and Pandey, 2018). *Fusarium solani* has a fast growth rate, higher capacity of metal ion reduction, nanoparticles formation and higher yield of biomass (Abd EL-Aziz et al., 2015), with higher tolerance to heavy metals like Cd(II), Cr(VI), Ni, Pb, Zn(II), Co(II), and Pb(II) (Vargas-García et al., 2012; El-Sayed, 2014; Rasha, 2017). The objective of this study was to assess the different tolerance mechanisms of *F. solani* to Ag(I), and to determine the optimum conditions for maximum absorption and transformation of Ag(I) by *F. solani.*

## 2 Materials and methods

### 2.1 Fungal isolate and silver tolerance assay

*Fusarium solani* KJ 623702 was isolated and identified as described in our previous study (El-Sayed, 2014; El-Sayed et al., 2012, 2013a, b), maintained on potato dextrose agar (PDA) as slope cultures at 4 °C. The PDA medium was prepared and amended with different concentrations of AgNO_3,_ shaken very well, to obtain the desired concentrations (0, 200, 400, 600, 800, 900, 1000, 1100, and 1200 mg/l), then poured into the plates. The medium was inoculated with 8 mm agar plugs from 6-day-old fungal colonies in three replicates. The plates were incubated for 7 days at 28 °C, the visual growth of the fungal cultures was monitored. The minimum inhibitory concentration (MIC) was expressed by the lowest concentration of Ag(I) inhibiting the visible growth of *F. solani.* The MIC considered as the highest metal concentration tolerated by the tested fungus (Sabatini et al., 2016).

### 2.2 SEM and TEM analyses

To evaluate the morphological changes caused by Ag(I)-stress, *F. solani* treated with sub-MIC concentrations of Ag(I) (1000 mg/l) for 7 days at 28 °C was inspected with SEM (El-Sayed and El-Sayed, 2020). Fungal mycelia were fixed in glutaraldehyde (2.5 %) at 4 °C for 24 h and post-fixed in 1.0 % osmium tetraoxide at room temperature for 1 h. The samples were dehydrated with acetone, coated with gold and checked by a Jeol scanning electron microscope (JEM-1200XII).

For viewing the cytomorphological alterations induced by Ag(I)-stress, *F. solani* treated with Ag(I) at the sub-MIC concentration (1000 mg/l) for 7 days at 28 °C was investigated with TEM Samples were dipped in 2.5% glutaraldehyde, primary fixative, for 3 h at 4 °C, rinsed with 0.2 M phosphate buffer (pH 7.4) for 30 min, then post-fixed in osmium tetraoxide (1.0 %) for 2 h at 4 °C and washed with phosphate buffer for 30 min (Kebeish and El-Sayed, 2012; El-Sayed et al., 2015a, b). For dehydration, samples then passed in a series of ethanol (50%–100%) and transferred through three changes of acetone: ethanol (1:2, 1:1 and 2:0) for 10 min each and embedded in epoxy medium (Epon 812). The sections were stained with uranyl acetate followed by lead citrate for 30 min. Transmission and photographing accomplished by a JEOL-1010 electron microscope (Regional Center of Mycology and Biotechnology, Cairo, Egypt).

### 2.3 Energy dispersive X-ray (EDX) microanalysis

To verify the bioaccumulation, *F. solani* was treated with Ag(I) at the sub-MIC concentration (1000 mg/l) for 7 days at 28 °C and examined with EDX microanalysis for semi-quantitative and qualitative elemental determination by an X-ray microanalyzer (model Oxford 6587 INCA X-sight) combined with a JEOL JSM-5500 LV scanning electron microscope at Regional Center of Mycology and Biotechnology, Cairo, Egypt.

### 2.4 Growth of *F. solani* in response to Ag(I)

Stock Ag(I) solution was prepared by dissolving 1 gm AgNO_3_ in 100 ml deionized water. To investigate the effect of Ag(I) stress on *F. solani*, the tolerance index (TI), biomass production, bioremoval percentage, lipid peroxidation, H_2_O_2_ content, total soluble protein, total antioxidant, and total thiol, and the activity of polyphenol oxidase (PPO) were determined. The tolerance index (TI), an indicator of the microorganism response to metal ions stress, was calculated from the fungal growth in presence of metals divided by the fungal growth in absence of metals under the same conditions (Anahid et al., 2011). The tolerance index of *F. solani* was assessed for Ag(I), comparing to the control culture (without silver ions). Eight mm diameter agar plugs from a 6-day-old culture was inoculated at the center of PDA plates amended with 200, 400, 600, 800 and 1000 mg/l AgNO_3_ then incubated at 28 °C for 14 days. The mean of perpendicular diameter measurements was calculated for every plate on the 14^th^day. The radial growth was determined from four measurements (mm) that crossed through the center of the inoculated portion. The TI was estimated according to: 0.00–0.39 (very low tolerance), 0.40–0.59 (low tolerance), 0.60–0.79 (moderate tolerance), 0.80–0.99 (high tolerance) and 1.00–>1.00 (very high tolerance) (Oladipo et al., 2018).

The percentage of Ag(I) removal and the effect of metal ions on the biomass of *F. solani* was evaluated. Sterilized solutions of AgNO_3_ were aseptically added to the sterilized PDB medium to get the final concentrations 10, 50, 100, 150, 200, 250, 300, 350, 400, 450, and 500 mg/l and incubated at 28 °C for 7 days at 120 rpm. AgNO_3_-free cultures of *F. solani* were used as control. During the fungal growth in presence of Ag(I), the color of culture media was changed into a brown color indicating the reduction of Ag(I) ions and formation of silver nanoparticles (AgNPs). The biomass was harvested by filtration and dried into a constant weight at 60 °C and the average dry weight was recorded. The culture filtrates were centrifuged to collect the particles of AgNPs prior to UV-Visible spectroscopic analysis (T80 UV–Vis spectrophotometer, Germany), transmission electron microscopy (TEM) (JEOL TEM-1400 electron microscope at Regional Center of Mycology and Biotechnology, Cairo, Egypt), and X-ray powder diffraction (XRD) a Broker D8 Advanced target Cu Koα powder diffractometer (λ = 1.5418 A°) over the range of 0–60 2θ, at Central Metallurgical & Development Institute, Helwan, Egypt. For TEM analysis, a drop of the sample placed on the carbon coated grid of the microscope. For XRD, the samples were coated as a thin film on glass slides, dried at 50 °C. The residual Ag(I) was estimated using an atomic adsorption spectrophotometer (Model Unicam 969).

The percentage of metal removal was determined according the following equation:

Removal(%)= (C_i_ − C_f_)/C_i_ × 100

Where, C_i_ = initial concentration of Ag(I) (mg/l); C_f_ = residual concentration of Ag(I) (mg/l).

The influence of Ag(I) on the total thiol content, total antioxidant, total soluble protein, and activity of polyphenol oxidase (PPO) and catalase (CAT) by *F. solani* were determined. The mycelia were homogenized with 50 mM cold phosphate buffer (pH 7.0) of 50 mM EDTA using a chilled mortar, centrifuged at 6000 rpm for 15 min at 4 °C. The supernatants and culture filtrates were used to elucidate the tolerance mechanism of *F. solani* to Ag(I).

### 2.5 Polyphenol oxidase (PPO) activity

Polyphenol oxidase (PPO) activity was evaluated according to Bergmeyer et al. (1974). The reaction mixture contains enzyme preparation (200 μl) in 0.1 M potassium phosphate buffer at pH 7.0, 0.5 U/ml horseradish peroxidase, guaiacol (0.2 mM), and catechol (10 mM) in a total volume 1 ml. The reaction mixture was incubated for 60 min at 30 °C and then frozen for 10 min. The developed color was measured at 436 nm. The activity of PPO was expressed by the amount of enzyme releasing 1 μmol H_2_O_2_ per min/mg protein/min.

### 2.6 Total antioxidant, total thiol and total protein contents

The total antioxidant concentration of the fungal extract was evaluated by the ferric-thiocyanate assay (Gupta et al., 2004; El-Sayed et al., 2013, 2014). Briefly, the crude fungal preparation (1 ml) was mixed with 30% ammonium thiocyanate and 20 mM ferrous chloride, then incubated for 10 min. The developed red color was recorded at 500 nm.

The total thiol contents of the fungal extracts were determined by Dithionitrobenzoic acid (Ellman, 1959, El-Sayed et al., 2016, 2021; 2015c). Briefly, the reaction contains 3 μl of the fungal extract, 20 μl of 0.01 M DTNB, shaken very well and absorbance was measured at 412 nm.

The total protein content was measured by Folin's reagent (Lowry et al., 1951) using bovine serum albumin as standard.

### 2.7 Hydrogen peroxide (H_2_O_2_), malonyl dialdehyde (MDA), and oxalic acid contents

Hydrogen peroxide (H_2_O_2_) content was determined according to the method of Alexieva et al. (2001). One gram of mycelium was homogenized in 0.1 trichloroacetic acid and filtered by Whatman No. 1 filter paper. Two milliliters of the reagent (1 M KI in distilled water) and 0.5 ml 100 mM potassium phosphate buffer (pH 6.8) was added to 0.5 ml of the mycelium extract. The reaction was incubated for 1 h in dark, stopped with 0.1% TCA, and the absorbance was measured at 390 nm. The amount of H_2_O_2_ was calculated from the standard curve of authentic concentrations of H_2_O_2_, and expressed by μg/g fresh weight.

The total malonaldehyde (MDA) concentration was determined according to Li (2000). The fungal mycelia (0.2 g) homogenized in 5% TCA (1.5 ml) and the homogenate was centrifuged at 6000 rpm for 20 min. The reaction mixture contained 0.5 ml mycelial extract, 1ml of 20 % TCA, and 1 ml of 0.5 % thiobarbituric acid, incubated at 95 °C for 25 min, then cooled immediately, centrifuged, and the absorbance was determined at 450, 532 and 600 nm, respectively.

MDA(μg/ml) = 6.45 (A532- A600)-0.54 A450

To investigate the role of oxalic acid in Ag(I) tolerance, the concentrations of oxalic acid in Ag(I)-free (control) and Ag(I)-stressed culture filtrates (400 mg/l) were determined with HPLC. HPLC system comprised a GBC UV/vis detector, GBC LC 1110 pump controlled by WinChrome chromatography (Kromasil column, 100 × 4.6 mm). The eluent was 85 % acetonitrile:15% water at flow rate 1 ml/min. The samples of oxalic acid were determined by measuring the absorbance at 254 nm comparing to authentic concentrations of oxalic acid.

### 2.8 Biosorption and FTIR and EDX analyses

To explore the relationship between tolerance and biosorption potential of *F. solani* and Ag(I), the batch biosorption studies was carried out. *F. solani* was cultured on PD broth, incubated at 28 °C for 7 days at 120 rpm, the fungal biomass was separated by filtration and then washed with sterile distilled water. A comparison between live (not growing) and alkali-treated biomass was studied. One part of the biomass was directly used in metal uptake experiments to evaluate the metal removal in terms of the biosorption capacity of living (not growing) biomass. The second part was alkali treated by mixing the biomass with 0.2N NaOH for 1 h then washing till the pH reached to neutral (6.8–7.2) (Kapoor and Viramghavan, 1998).

The biosorption experiments were performed in 250 ml Erlenmeyer flasks containing 100 ml of Ag(I) solution. The effect of different pH (2–6), biosorbent dose (0.5–2.5), initial metal ion concentration (50–350 mg/l), contact time (0.08–24h), and temperature (5–55 °C) on the biosorption was determined. The mixtures were agitated at 150 rpm. All the experiments were run in triplicates. The biosorbent was then separated from the solution by centrifugation at 6000 rpm for 15 min. The residual Ag(I) concentration in the supernatant then was assessed as described above.

q = C_i_-C_f_ /m × V (Fan et al., 2008)

Where q is the biosorption capacity (mg/g), C_i_ is the initial Ag(I) concentration (mg/l), C_f_ is the final metal ion concentration (mg/l), M is the mass of the biosorbent (g), and V is the volume of metal solution.

The Ag(I)-free living (NU) and alkali-treated (TU) biomass (control) and Ag(I)-loaded living (NL) and alkali-treated biomass (TL) were characterized by FTIR (PerkinElmer FTIR 1650, Center of Microanalysis, Cairo University, Egypt) and EDX. FTIR studies were recorded over the region 400–4,000 cm^−1^ to elucidate the functional groups involved in the biosorption. For EDX and FTIR studies, biomass were subjected to optimum biosorption conditions at initial pH 5, initial Ag(I) concentration 200 mg/l, biosorbent dose 0.5 g/l, contact time 1 h (native biomass) and 3 h (alkali-treated biomass), temperature 35 °C at 150 rpm.

## 3 Results and discussion

### 3.1 Silver tolerance of *F. solani*

Morphological, physiological, and biochemical adaptation strategies by microorganisms to resist heavy metals toxicity is nominated as metal tolerance (Mohammadian Fazil et al., 2015). *F. solani* showed tolerance to Ag(I) concentration up to 1100 mg/l which is 220,000-fold over the maximum acceptable limit permitted in drinking water (0.005 mg/l) (WHO, 2002). This value was very high as compared with that evaluated for *Fusarium* spp. (Xu et al., 2009). Highly tolerant fungi to toxic metals may be helpful in metal remediation. On the other hand, Zafar et al. (2007) concluded that there was no correlation between tolerance and biosorption of chromium and cadmium by *Rhizopus* sp. and *Aspergillus* spp. due to the differences in the mechanisms of resistance and biosorption. During the growth of *F. solani* at concentrations >200 mg/l of Ag(I), the media under the fungal growth turned brown, assuming the ability of strain to degrade AgNO3 by utilizing it as nitrogen source and the creation of AgNPs. The control (Ag(I)-free) plates did not show discoloration. At >800 mg/l of Ag(I), the growth pattern of *F. solani* was changed from loose hyphae with peripheral nature to densely entangled compact hyphae, allocated at the interior of colony and raised upward. Ban et al. (2012) observed curling hyphae with the formation of hyphal coils in *Gaeumannomyces cylindrosporus* in response to lead ions. *Penicillium* spp. were screened for tolerance to heavy metals on Czapek Dox Agar medium containing different concentrations of lead, copper, and cadmium. The significant variations in the cultural and morphological features were reported. The different responses depended on the resistance mechanisms of the organism and on the toxicity of the metals that in turn is affected by the concentration and the form of the heavy metal (Nazareth and Marbaniang, 2008). Pervez et al. (2009) attributed the strong increase on hyphal density of *Pleurotus ostreatus* in presence of cadmium to the increased number of laterals branches, with the decrease in the distance between branch points. *F. solani* exhibited a high tolerance to Ag(I)at 200, 400, 600 and 800 mg/l of TI value of 0.95, 0.91, 0.87, and 0.80, respectively, and very low tolerance to Ag(I) at1000 mg/l (TI, 0.31). The reduced TI reflects the inhibitory growth function of heavy metals (Ge et al., 2011).

### 3.2 SEM, EDX and TEM analyses

**Figure 1:**
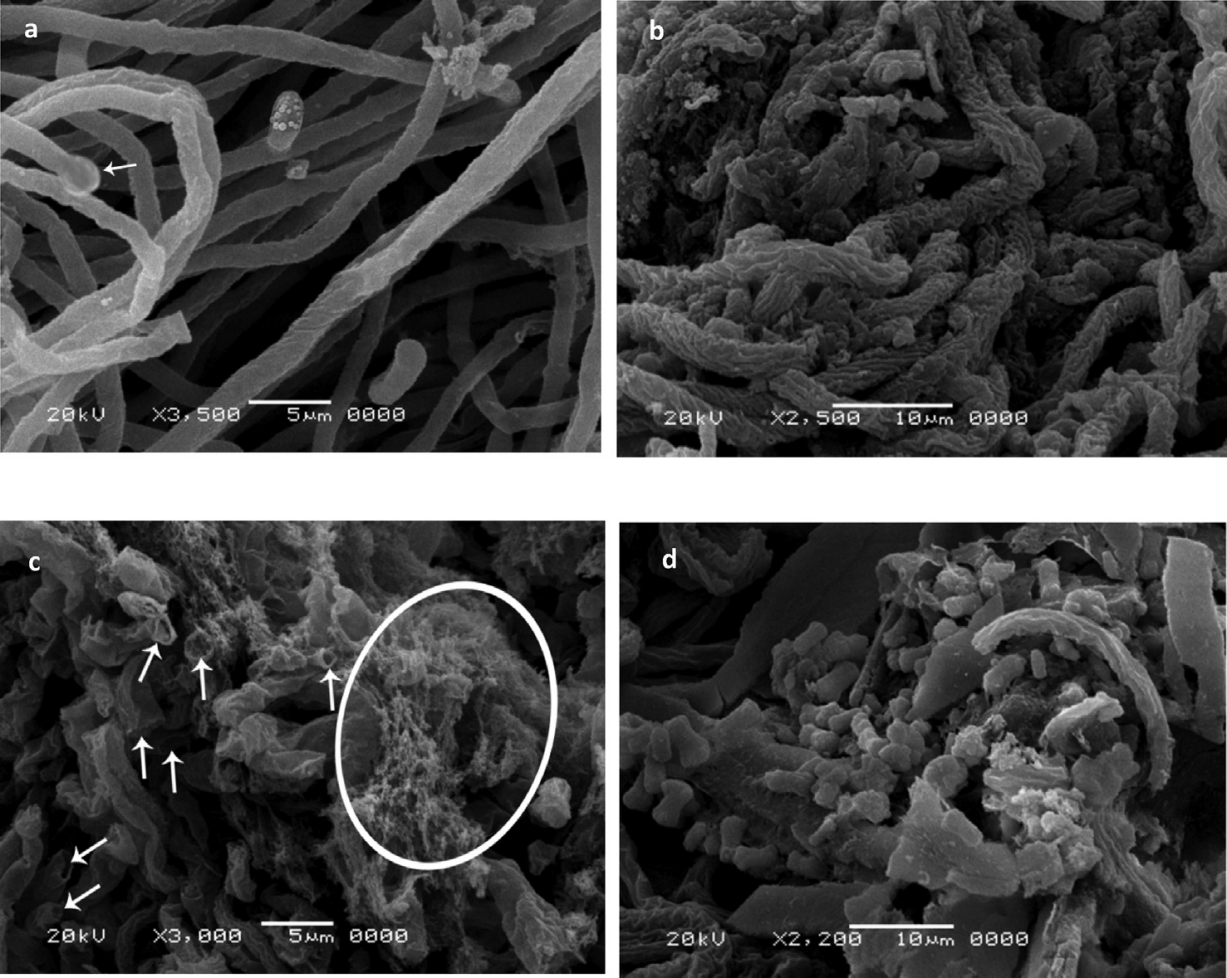
SEM micrograph of growing *F. solani* (a) Ag(I)-free pellets (control), (b–d) Ag(I) -loaded pellets (1000 mg/l). *F. solani* cultures were incubated for 7 days at 28 °C.

SEM micrographs of the growing biomass before and after Ag(I) exposure shown in Figure 1a-d. The surface of the unloaded mycelia was smooth. Microconidia and macroconidia were present (Figure 1a). Ultrastructural analysis revealed alterations in the density of mycelia, the texture of the surface and the diameter of hyphae in loade1d samples (Figure 1b). Moreover, the mycelia became wrapped by a mass of a gelatinous substance with inhibition in the formation of conidiogenous cells (Figure 1c) with complete damage (Figure 1d). Conidiogenous cells are responsible for microconidia and macroconidia formation (Sempere and Santamarina, 2009). When the toxic metals enter the fungal spores, they associate with the particulate insoluble cytoplasmic components and react with cytoplasmic receptor sites and caused inhibition of spore germination (Nazareth and Marbaniang, 2008). Fundamentally, the exposure to metal stress induced structural changes associated with the type of metal and its concentration (Kim et al., 2012; Luna et al., 2015). The morphological changes may be essential for metal removal (Li et al., 2017; Gururajan and Belur, 2018). The extracellular metabolic products complex Ag(I) through precipitation, ion exchange, or cell-surface binding (Jiang et al., 2016).

**Figure 2:**
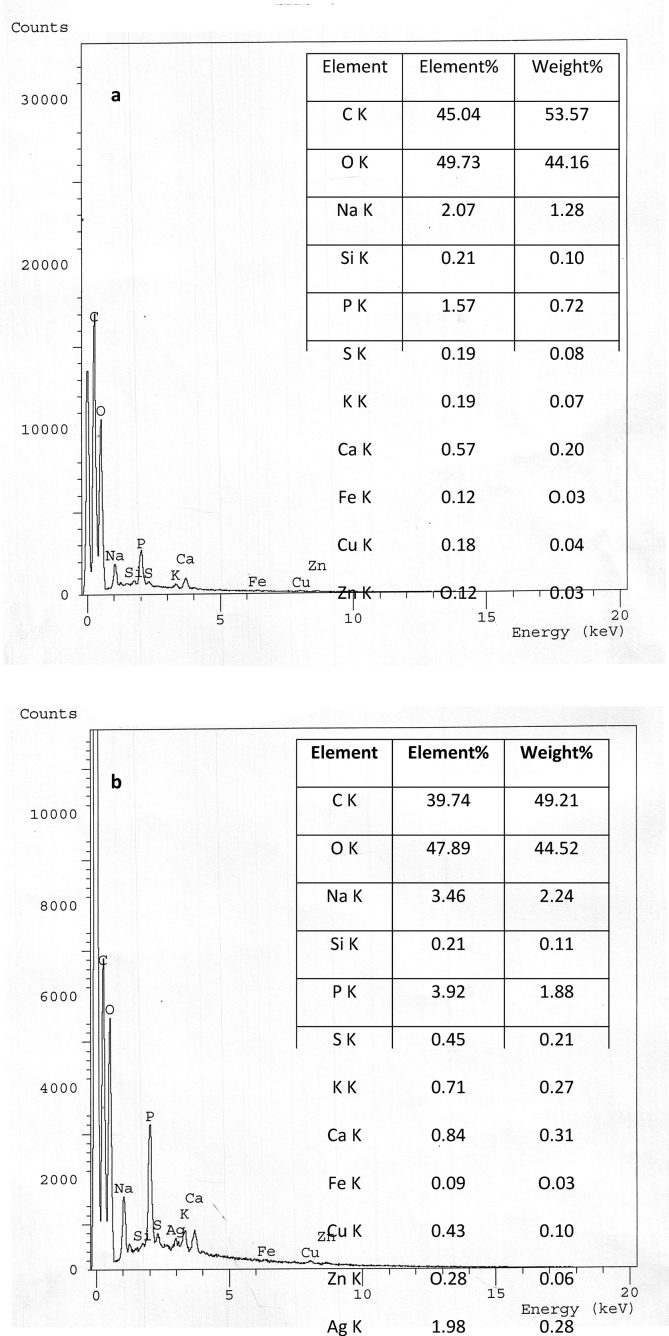
EDX of growing *F. solani* (a) Ag(I)-free pellets (control), Ag(I) -loaded pellets (1000 mg/l). *F. solani* cultures were incubated for 7 days at 28 °C.

The EDX spectra of control biomass did not reveal any signal for the presence of metal (Figure 2a). The Ag(I) signals on the metal-loaded biomass confirmed the ability of *F. solani* to bioaccumulate and precipitate Ag(I) (Figure 2b). An increase in percentage of element of K, P, S, Cu, Na, and Ca by 3.74, 2.5, 2.39, 2.37, 1.67, and 1.47folds the control, respectively, were recorded, as consistent with those reported by Gururajan and Belur (2018). An increase in element% of some metal ions after uptake of Ag(I) may be due to the involvement of ion exchange mechanism and/or the formation of silver complexes to reduce Ag(I) toxicity. Ag(I) has a high affinity to thiolates and binds strongly to sites ordinarily occupied by Cu(I). The copper efflux ATPase of *Enterococcus hirae* was shown to pump Ag(I) with the same velocity and affinity as Cu(I) (Solioz et al., 2011). Under heavy metal stress, an increase in cysteine synthesis, sulfate assimilation was observed. Also, the release of phosphorus that sequesters and chelates heavy metal ions occurred (Lima et al., 2013).

**Figure 3:**
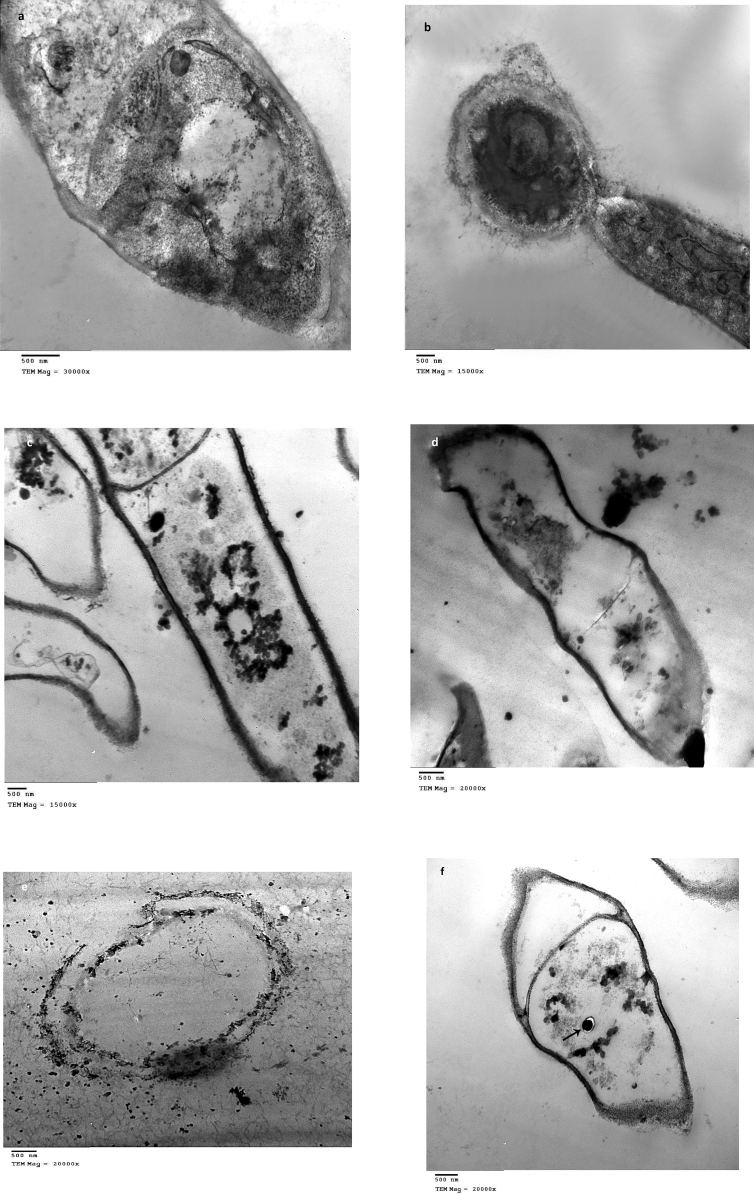
TEM of growing *F. solani* (a and b) Ag(I)-free pellets (control), (c–f) Ag(I) -loaded pellets (1000 mg/l). *F. solani* cultures were incubated for 7 days at 28 °C.

To show the cellular localization of bioaccumulated Ag(I), *F. solani* biomass subjected to transmission electron microscopy (TEM) (Figure 3a-f). Ultrathin sections of metal-free control cells showed a discrete cell wall (100 nm) and clear cytoplasm with normal organelles. Septum thickness was found to be 50 nm (Figure 3a). Conidia formation was visualized in Figure 3b. Ag(I)-loaded cells exhibited dark and relatively thicker cell wall (150 nm) and internally distributed silver complexes (Figure 3c). The wall is a highly effective structure and is capable to adapt to several changes. In *F. solani*, the relative thickening of the wall is probably due to an increase in chitin synthesis (Nazareth and Marbaniang, 2008). Extracellular metal complexes are found very close to cells. Septa became very thin (Figure 3d). Exopolysaccharides and other metabolites immobilize heavy metals to prevent their entry to the cells (avoidance mechanism) (García-Hernández et al., 2017; El-Sayed et al., 2017). Electron dense particles were distributed outside the cell, on the cell wall and plasma membrane and some were located within the cytoplasm, revealing the main interaction occurred in the cell wall (Figure 3e). Vacuolation was very clear (Figure 3f), intracellular bioaccumulation and chemical transformation to diminish metal in the cytosol (Saha and Orvig, 2010), was clearly observed. In some Ag(I)-loaded cells, there was lysis to the cell wall, plasma membrane, and internal organelles. This distortion may be due to the oxidative stress of Ag(I). Attachment of Ag(I) to the surface of the cell membrane causes an interruption in its selective permeability and the metabolic pathways of the cell. Besides, Ag(I) binds to the DNA, interjects between base pairs, and denatures the DNA, preventing its replication (Adriano et al., 2018).

### 3.3 Effect of Ag(I) on the growth of *F. solani*

The growth of *F. solani* on solid media did not follow the same pattern on liquid media, that might be due to formation of heavy metal gradients in agar that giving a protective chelating effect (Maizel et al., 2016). Therefore, tolerance of *F. solani* to silver ions on PDB is partially different from PDA. Many researchers have observed good results in metal bioaccumulation by applying fungi but nearly all have used submerged cultures along with Sabouraud Dextrose Broth (Zafar et al., 2007; Luna et al., 2015), Malt Extract Broth (Cecchi et al., 2017), Czapek Dox Broth Ban et al. (2012) which is more costly in comparison with PDB. By using potatoes, PDB can simply be prepared in the laboratory (Zhan et al., 2015; Bano et al., 2018). The biomass of *F. solani* was slightly decreased (8.4%) at 10 mg/l Ag(I), while at concentration ≥50 mg/l Ag(I), the fungal growth was progressively impaired, with mycelial darkening (Figure S1). The biomass was dramatically reduced by about 71.0–92.5% at 250–450 mg/l Ag(I) compared to control, with no growth at 500 mg/l. Heavy metals cause pose deleterious effects on microorganisms through the generation of ROS, influencing the formation of DNA and protein adducts, inhibition of enzymatic functions (competitively or non-competitively) and (Gauthier et al., 2014), and adhering to the cell surface and cause ion imbalance (Chen et al., 2014). The percentage of removal of Ag(I) was increased with the increasing concentrations of Ag(I) from 10 mg/l to 250 mg/l (17.3–73.4%), suggesting the uptake dependence on biomass and Ag(I) dose (Figure S2). At 350 mg/l Ag(I), the biomass was declined drastically, the percentage of removal was decreased by about 42.1%. Consistent results ensuring the enhancement of fungal metabolism to sustain its cellular process to higher concentrations of Ag(I) stress was reported for *A. alliaceus*, *T. harzianum,* and *Clonostachys rosea* (Cecchi et al., 2017).

The primary detection of AgNPs was carried out by visual observation of color change of the culture filtrate of *F. solani* starting at ≥150 mg/l of Ag(I). The biosorbed metal ions can be transported into the microbial cells metabolism-dependently where redox state of metal ions possibly altered to reduce their toxicity (Yin et al., 2019).

**Figure 4:**
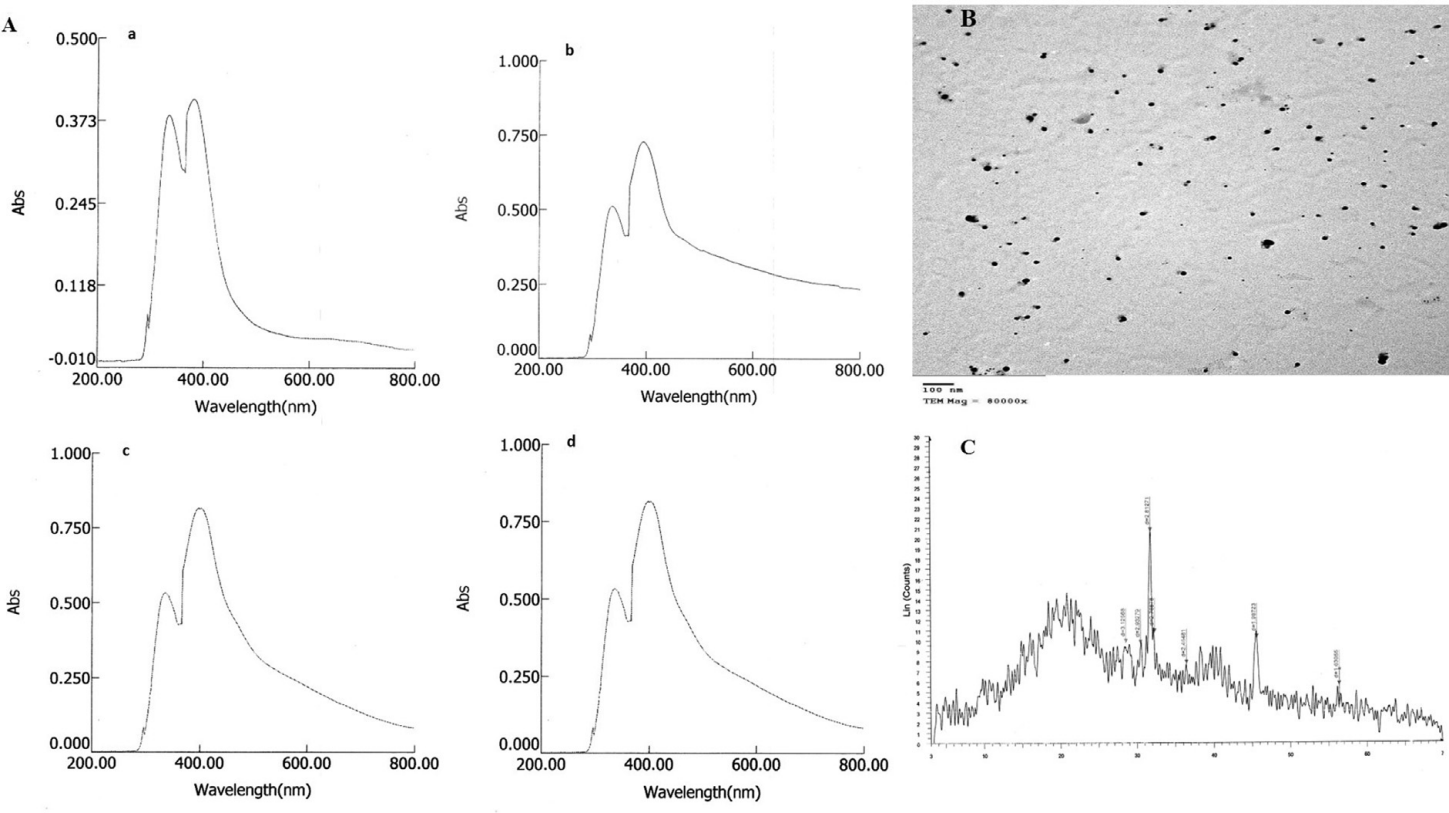
A. UV-Visible spectra of silver nanoparticles synthesized by *F. solani* in the presence of (a) 250 mg Ag(I)/l, (b) 300 mg Ag(I)/l, (c) 350 mg Ag(I)/l, and (d) 400 mg Ag(I)/l. B. TEM image of silver nanoparticles synthesized by *F. solani* in the presence of 300 mg Ag(I)/l. C. XRD pattern of silver nanoparticles synthesized by *F. solani* in the presence of 300 mg Ag(I)/l.

The surface plasmon resonance (SPR) is a profound criterion in determination of optical absorption spectra of metal nanoparticles. The UV absorption bands of CF occurred at 372 nm (250 mg/l Ag(I)), 382 nm (300 mg/l Ag(I)), 394 nm (350 mg/l Ag(I)) and 402 nm (400 mg/l Ag(I)) (Figure 4A). The position and width of SPR absorption peak depend on the size of NPs (Jeyasundari et al., 2017). The intensity of the absorption peaks was increased from 0.33 to 0.82 OD with the increase of Ag(I) concentration. The diameters of spherical AgNPs were ranged from 10.25 to 21.19 nm (15.22 ± 3.14 nm average size) (Figure 4B). The particles were confirmed as elemental Ag(0) using XRD (Figure 4C).

### 3.4 PPO and CAT activity

The PPO and CAT activities by *F. solani* were induced in presence of Ag(I) (Figure S3). Compared to 10 mg/l Ag(I), the PPO activity was enhanced by 113.9 % at 150 mg/l Ag(I), completely inhibited at 250 mg/l. CAT activity was increased by 45.4% at 200 mg/l and completely inhibited at 250 mg/l. The decrease in CAT and PPO activities may be due to the inhibitory effect of released ROS in response to Ag(I). The activities of antioxidant enzymes by *F. solani* were increased consequently responsive to metal stress till maximum value, followed by a subsequent decrease with the higher heavy metal concentration (Kusvuran et al., 2016)*. A. niger* had both non-enzymatic and enzymatic protective antioxidant mechanisms in response to copper stress (Luna et al., 2015). A strong increase in total antioxidant (232.4–456.3 μM) was recorded at concentrations from 100-300 mg/l Ag(I), followed by a sudden decrease at 350 mg/l of Ag(I)/l (154.6 μM) and complete inhibition at 400 mg/l. The increasing on the antioxidant activity in response to heavy metal stress has been frequently reported for various fungal isolates (Azevedo et al., 2016).

### 3.5 Effect of Ag(I) on thiol content of *F. solani*

Thiol compounds are the prime agents for heavy metal tolerance, for retaining the redox homeostasis of the cells (García-Hernández et al., 2017). Thiol contents by *F. solani* was increased gradually with the stress of Ag(I) during the growth (Figure S4). The highest intracellular and extracellular thiol by *F. solani* were 170.2 and 244.4%, respectively, compared to control at 250 mg/l. Beyond this concentration, the progressive decrease of thiol contents was observed. The increased levels of thiol after metal exposure confirmed their importance in fungal survival. Some members of the thiol family are capable of binding heavy metal ions via thiolate coordination in fungi (Ban et al., 2012; Azevedo et al., 2016).

### 3.6 Effect of Ag(I) on protein content of *F. solani*

Synthesis of binding and metal transport proteins are one of the tolerance strategies of microorganisms to heavy metals (Baldrian, 2010). Variations in the soluble proteins in *F. solani* under different concentrations of Ag(I) were studied (Figure S5). An initial increase on intracellular and extracellular proteins were observed with Ag(I) treatments, that reached to the maximum values at 300 mg/l Ag(I) 113.32% and 181.73 %, respectively, compared to control. Therefore, accumulation of Ag(I) at non-lethal concentrations at the cell surface leads to the unrestricted flow of nutrients into cells and stimulate the metabolic activity (Ahonen-Jounarth et al., 2004). Total soluble protein contents were greatly reduced at 400 mg/l Ag(I), that might be a consequence of intolerance to high Ag(I). Guelfi et al. (2003) attributed the reduction in protein contents of *A. nidulans* to the autolysis of mycelium and subsequent proteolytic breakdown in the presence of high concentrations of cadmium.

### 3.7 Physiological response of *F. solani* to Ag(I)

Excess Ag(I) induced oxidative stress and generated ROS. ROS reacted with the methylene groups of the polyunsaturated fatty acids of plasma membrane, causing lipid peroxidation and formation of MDA (Vasilaki and McMillan, 2011). MDA and H_2_O_2_ contents (Figure S6) were increased gradually 28.6%–142.8% and 1.26-to 3.84-fold, respectively, for 10–300 mg/l Ag(I) treatments. Cadmium stress appreciably stimulated the production of H_2_O_2_, MDA, and superoxide anion in the mycelia of a dark septate endophyte *Exophiala pisciphila* (Zhan et al., 2015).

**Figure 5:**
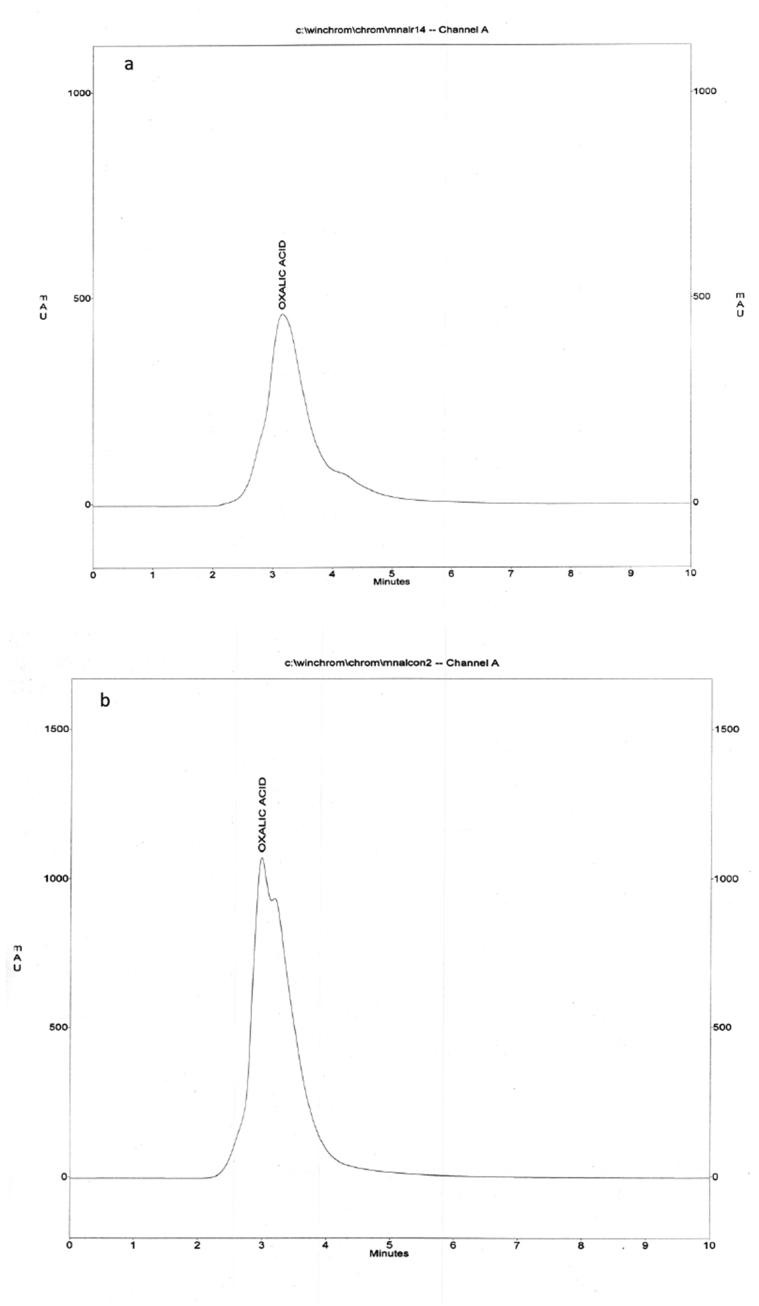
HPLC chromatograms of *F. solani* (a) Ag(I)-free culture filtrate (control), (b) Ag(I)-supplemented culture filtrate (400 mg/l).

Oxalate is a typical metal chelator excreted by fungi. The oxalate anion can immobilize and detoxify metals by formation of insoluble metal oxalate complexes (Gadd et al., 2014). HPLC chromatograms of Ag(I)-free control and Ag(I)-stressed samples (400 mg/l) showed that oxalic acid concentrations were 270 μg/ml and 1197.67 μg/ml, respectively, (Fig. 5a and b). Ag(I) stimulated the production of oxalic acid (343.58%, as compared to control). Extracellular oxalic acid, Extracellular biosorption, and intracellular bioaccumulation played principal roles in the tolerance of lead by *P. ostreatus* (Wang et al., 2019).

### 3.8 Physical factors affecting the biosorption of silver ion by *F. solani*

Environmental factors controlling the biosorption include concentration, and oxidation state of the metal ions, temperature, pH of wastewater, character and concentration of the biosorbent, and mechanism of metal removal. The combined effects of these parameters influence the metal speciation (Kanamarlapudi et al., 2018).

The initial pH of the solution is the most critical factor in the biosorption, influencing the activity and amount of the biosorbent functional groups such as carboxylate, phosphate, and amino groups, and the chemical characters of metal ions (Feng et al., 2018). The extent of adsorption relies on either the respective charges on the biosorbent and cations or the fundamental formation constants of the complexation reactions (Al-Hamdan and Reddy, 2006). The effect of initial pH ranged from 2–6 was assessed to prevent the formation of AgOH precipitate. Ag(I) is a weak Bronsted acid has an exponent of the acid dissociation constant - pKa = 11.7. Hence, the precipitation of AgOH occurred at relatively high pH in the range 6.5–7.5 (Charlot, 1969). The Ag(I) removal capacities of native and alkali-treated biomass were low at pH 2.0 (5.19 % for native biomass and 4.25 %for treated biomass) (Figure S7). At low pH, protonation of the cell wall groups and the competition between oxonium (hydronium H_3_O^+^) ions and metal ions was occurred. The Ag(I) removal capacities was increased with the increase on pH value up to 5.0 (138.9% for native and 75.23% for treated biomass). Above pH 3.0, the glycoprotein of the cell wall, chitin, proteins gained many phosphate and carboxyl groups that are known to play a chief role in biosorption (Mahmoud et al., 2017). The sudden increase in biosorption with a small increase in pH value is often referred to an adsorption edge (Varshney et al., 2011). As the pH increased, the concentration of H_3_O^+^ ions was decreased and deprotonation of binding sites was observed. As a result, the competing effect of H_3_O^+^ ions was reduced and exchange of H^+^ with the metal ions was induced (Mrudula et al., 2016). The decrease in biosorption above pH 5.0could be attributed to the speciation of the metal ions and the formation of AgOH that do not adsorbed well (Iram and Abrar, 2015). Similarly, the maximum biosorption ofiron by *A.niger*was obtained at pH 5.4 and above this pH, a decline in the uptake capacity happened as reported by Iram et al. (2015). Alkali treatments increase the electronegativity of the fungal biomass by the ionization of functional groups, thus attract more cations Bux and Kasan (1994); Renitta et al. (2019). A decrease in biosorption of Ag(I) in alkali-treated biomass was observed that may be due to the hydrolysis and disintegration of biomass by NaOH solution (Hu et al., 2015).

The initial metal ion concentration has a fundamental role as a driving force to cope with the mass transfer resistance between the solid and aqueous phases. With the increase on the metal ion concentration from 50 to 200 mg/l of Ag(I), the uptake capacities of *F. solani* was increased from 7.21 to 13.71 mg Ag(I)/g (native biomass) and from 5.11 to 8.9 mg/g (treated biomass) and reached saturation values (Figure S8). The biosorption capacities were then decreased with the rise in initial metal ion concentration to 350 mg/l. At low initial metal concentration, the proportion of the initial concentrations of solute to the available surface area was minimum, thus, the fractional biosorption didn't depend on their initial concentration (Binupriya et al., 2007). The maximum uptake capacity at 200 mg Ag(I)/l, was attributed to the high availability of metal ions and the chance of collision between the ions and biosorbent (El-Gendy et al., 2017). Reducing the uptake capacity at high metal concentration could be assigned to the competition between ions and the scarcity of available free binding sites (García-Hernández et al., 2017).

The biosorbent concentration presents a high contribution to the biosorption due to the strong dependence on the number of available binding sites on the biosorbent surface, and electrostatic interactions between biosorbent cells (Iskandar et al., 2011). The highest uptake capacities of native (14.1 mg/g) and treated biomass (9.9 mg/g) of *F. solani* for Ag(I) was observed at the lowest biosorbent dose (0.5 g/l) (Figure S9). This mainly related to a high concentration of metal ions compared to the available number of surface-active groups (Mahmoud et al., 2017). The uptake capacities were decreased from 14.1 to 7.5 (native biomass) and from 9.9 to 5.4 mg/g (treated biomass), while, the biomass concentration was increased from 0.5 to 3.0 g/l. At the high biomass concentration, the metal ions are insufficient for complete distribution over the available active sites. Also, biomass can exert a shell effect that protects the binding sites from being occupied by Ag(I) (Kanamarlapudi et al., 2018).

Temperature has a critical influence on the adsorption process as it affects (positive/negative) the biosorption of metal ions (Al-Homaidan et al., 2014). The metal uptake was increased gradually with the temperature increase (5–35 °C) reaching a maximum of 15.8 mg/g (native) and 10.3 mg/g (treated) at 35 °C (Figure S10). However, the biosorption capacity was decreased by 33.54% (native) and 30.1% (treated) at high temperature (55 °C). As the collision frequency between biosorbent and Ag(I) increased at 35 °C, the particles of Ag could be sorbed electrostatically on the biosorbent surface. The exothermic nature of some biosorption processes could reduce the biosorption capacity in some microorganisms (Park et al., 2010; Tahir et al., 2014).

Contact time plays a vital role in the efficient removal of heavy metals. The time required to attain maximum biosorption depends on the type of biosorbent, metal ion and their combination (Kanamarlapudi et al., 2018). The biosorption capacity of native biomass had been increased from to as the contact time increased from 5 to 60 min, and this followed by equilibrium (Figure S11). On the contrary, the biosorption of Ag(I) by treated biomass was slow and reached equilibrium within 180 h. The biosorption includes rapid and passive phase followed by a slower and active one. In the passive phase, the large numbers of the binding sites are vacant and available for biosorption and the physical adsorption or ion exchange was occurred. With an increase on time, the rate of biosorption was decreases due to increase in percentage saturation by metal ions remaining in the solution (Abdel-Ghani et al., 2009).

### 3.9 Spectroscopic characterization of fungal biomass

#### 3.9.1 FTIR

**Figure 6:**
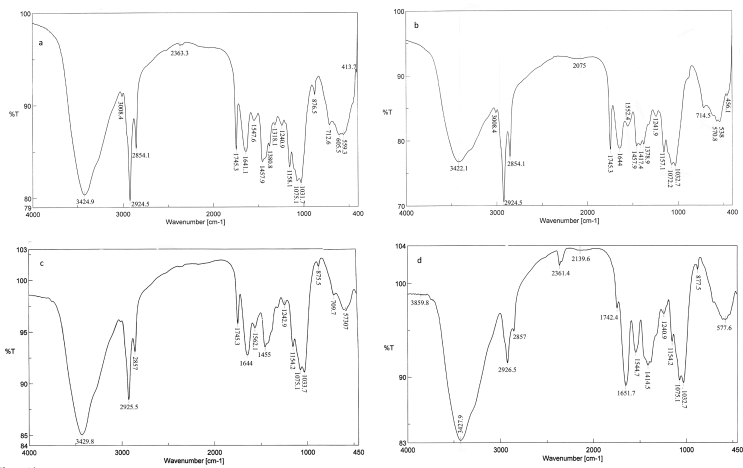
FTIR spectra of *F. solani*, (a) native cells, (b) Ag(I)-loaded cells, (c) alkali-treated biomass, and (d) Ag(I)-loaded alkali treated cells. Biosorption conditions: initial pH = 5, initial Ag(I) concentration = 200 mg/l, biosorbent dose = 0.5 g/l, contact time 1 h (native biomass) and 3 h (alkali-treated biomass), temperature = 35 °C at 150 rpm.

**Figure 7:**
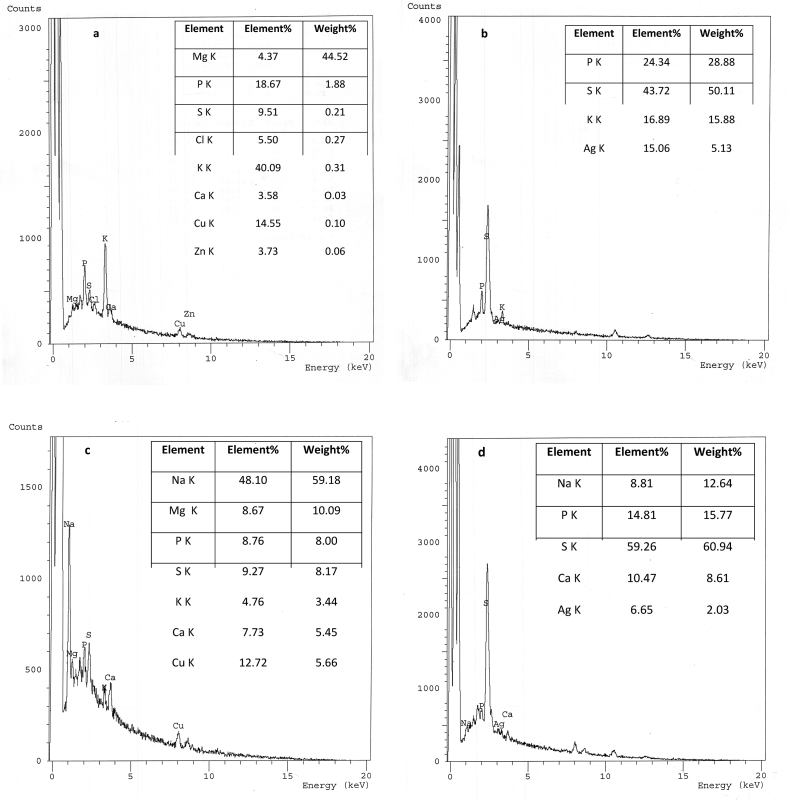
EDX microanalysis of *F. solani* (a) native cells, (b) Ag(I)-loaded cells and (c) alkali-treated cells, and (d) Ag(I)-loaded treated cells. Biosorption conditions: initial pH = 5, initial Ag(I) concentration = 200 mg/l, biosorbent dose = 0.5 g/l, contact time 1 h (native biomass) and 3 h (alkali-treated biomass), temperature = 35 °C at 150 rpm.

Biosorption of metal ions by the microbial cell wall is one of theremarkable interaction mechanisms. Therefore, many surface complexations models have been used to describe the extent of metal adsorption by microorganisms (El-Gendy et al., 2017). The FTIR spectra of the biomass of NU, NL, TU, and TL were represented in Figure 6a-d, respectively. The new absorption peak at 3859.83 cm^−1^in the case of TL biomass (Figure 6d) showed the role of alcohol groups in uptake. The shift in the waven umber around 3425 cm^−1^ revealed the interaction of an –NH2 asymmetric stretching mode of amines and –OH groups with Ag(I) uptake (Gururajan and Belur, 2018). Lead ions bioremoval by *Rhizopus nigricans* was found to be mainly due to the binding of lead to the amine-N of chitin, which then served as a nucleation site for the extra precipitation of lead (Zhang et al., 1998). The new intense peaks at 2361.41 and 2139.63 cm^−1^ (TL biomass) were representative to alkanes and C=O and C=N groups, respectively. The new band at 2075.03 cm^−1^in the case of NL biomass (Figure 6b), the shift at 1743.2 cm^−1^(TL biomass) and 1641 cm^−1^are due to the C=O stretching mode of carbonyl groups in esters, alcohol, ethers and carboxylic acids (Jeyasundari et al., 2017). The marked shift at 1562.06 cm^−1^(Δ 18 cm^−1^) (TL biomass) was due to the C–N stretching vibration, N–H bending vibration, and the complexation with N–H group (Hu et al., 2015). This shift indicated that acidic groups; carboxyl and hydroxyl, are the chief agents in uptake (El-Gendy et al., 2017). The shift at 1547.59 cm^−1^ (Δ 5 cm^−1^, NL biomass) can be attributed to the role of –CH, amide I and II in the process (Feng et al., 2018). A marked shift at 1455.03 cm^−1^ (Δ 41 cm^−1^) in case of TL biomass assigned to C–H bending in CH3 groups (scissoring)/aromatic –C=C stretching vibrations (Nikolic et al., 2010). A new peak at 1417.42 cm^−1^ (NL biomass) was assigned to –COOH group, deformation vibration of –OH, and the stretching vibration of C=O (Hu et al., 2015). Carboxyl, and secondary amines had a major role in binding and biotransformation of Ag(I) by *Cochliobolus lunatus, Lactococcus lactis,* and *L. casei* (Salunkhe et al., 2011 and Milanowski et al., 2017). The role of amide III, sulfonamide, sulfonyl, and C(O)–O stretching vibrations identified in the shift at 1376.78 cm^−1^ and the disappearance of peak at 1318.11 cm^−1^ (NL biomass). Moreover, there was a decrease in the peak intensity and a slight shift at 1239.97 cm^−1^ (Ar–O stretching) and 1158.04 cm^−1^ (C–O stretching) in NL biomass. The shift at1075.12 and 1031 cm^−1^ indicated the interaction of Ag(I) with sulfoxides, S=O stretching, sulfones, sulfonic acid, and sulfonamides. Moreover, the shift at 1040–1150 cm−1 may be due to the attachment of heavy metals to phosphate groups (Mahmoud et al., 2011). El-Gendy et al. (2017) characterized phosphorus compounds, C–N stretching, O–H bending, and sulfur compounds in the region between 1000–1400 cm^−1^. Disappearance and shift of bands after Ag(I) uptake by NL and TL biomass at 876.49 and 874.52 cm^−1^, respectively, indicated the intervention of P=S stretching and phosphorus in the process. Consistent with these results, EDX spectra of NL (Figure 7b) and TL (Figure 7d) showed a marked increase in element % of P and S by 37.75, 413.75, 69.06, and 539.27%, respectively. The spectra of EDX and FTIR indicated the involvement of cellular sulfur and phosphorus compounds in Ag(I) binding. A very marked shifts at 605.54 cm^−1^ (Δ 35 cm^−1^) and at 559.26 cm-1 (Δ 21 cm^−1^) (NL biomass) and at 573.72 cm^−1^ (TL biomass) were indicative of C–S stretching. The role of C–S stretching appeared again in a significant shift band at 413.93 cm^−1^ in the case of NL biomass (Δ 43 cm^−1^). Wang and Chen (2006) mentioned that soft metals, such as Ag(I), Cd (II), Pb(II), and Hg(II) form stable bonds with sulfur-containing (soft) ligands, nitrogen-, S−, SH−, CN−, R–NH2−, and imidazole. The greater the covalent index (X^2^_m_r) (where X_m_ is electronegativity and r is the ionic radius), the greater the potential to form covalent bonds with biological ligands in order S > N > 0 (Chen and Wang, 2007). The electronegativity and the ionic radius of Ag(I) are1.93 and 144 pm, respectively. The covalent index of Ag(I) is 5.36. Li et al. (2013) reported that that the sulfur compound was involved in Se(IV) biosorption by *Aspergillu*s sp. The bands at 2924.52, 2854.13, 1745.26, and 1457.92 cm^−1^ (NL biomass) and at 2854.02, 1124.19, and 1075.12 cm−1(TL biomass) showed no change after uptake of Ag(I).

After Ag(I) uptake, the total shifts in the case of NL biomass (Δ 104 cm^−1^) were more pronounced than in TL biomass (Δ 71 cm^−1^). This may interpret the high biosorption capacity of NL biomass. This in agreement with EDX elemental analysis which revealed that the elemental ratio % of Ag(I) was 15.06 (NL) and 6.65 (TL). In NL biomass, the chief shifting of peaks was in the region between 400-700 cm^−1^ and at 1547.59 cm^−1^ which attributed to C–S stretching. In TL biomass, the main functional groups involved were C=O and C=N groups, C – H bending, the C–N stretching vibration, and N–H bending vibration and the complexation with the N–H group. From EDX microanalysis signals for Cu(II) and Zn(II) completely disappeared after biosorption. Usually, the release of these metal ions from biosorbents in binding metal ions was regarded as an indicator of the mechanism of ion exchange for heavy metal binding (Reddad et al., 2002).

## 4 Conclusion

Fungi are the most frequently utilized organisms for bioremediation of toxic heavy metals that could be via biosorption and biotransformation, however, there a few studies explaining the mechanisms of heavy metals removal by fungi. Thus, *F. solani* has been used as model fungus to elucidate the mechanism of silver ion resistance and biotransformation. The fungal growth, bioaccumulation, organic acids, total non-proteineous antioxidants, and antioxidant enzymes of *F. solani* in response to silver ions were investigated. It has been plausibly noticed that oxalic acid of *F. solani* was increased by 3.5 folds in response silver ions comparing to control. Also, the level of Ag ions biosorption is highly dependent on treatment of biomass, pH values, initial metal ion concentrations, temperatures, and contact time.

## Declarations

### Author contribution statement

Manal T. El Sayed: Conceived and designed the experiments; Performed the experiments; Analyzed and interpreted the data.

Ashraf S.A. El-Sayed: Contributed reagents, materials, analysis tools or data; Wrote the paper.

### Funding statement

This work was supported by the Botany and Microbiology Department, Faculty of Science, 10.13039/501100007102Zagazig University, Egypt.

### Competing interest statement

The authors declare no conflict of interest.

### Additional information

No additional information is available for this paper.

## Appendix A Supplementary data

Supplementary Figures Heliyon April-26-2020 V2 Supplementary Figures Heliyon April-26-2020 V2
