# Supplementary material for: Tolerance and mycoremediation of silver ions by Fusarium solani
Source: Heliyon. 2020 May 12;6(5):e03866. doi: 10.1016/j.heliyon.2020.e03866 (PMC7225397; doi:10.1016/j.heliyon.2020.e03866)
Supplement: Supplementary Figures Heliyon April-26-2020 V2 [file mmc1.pptx]

## Slide 1
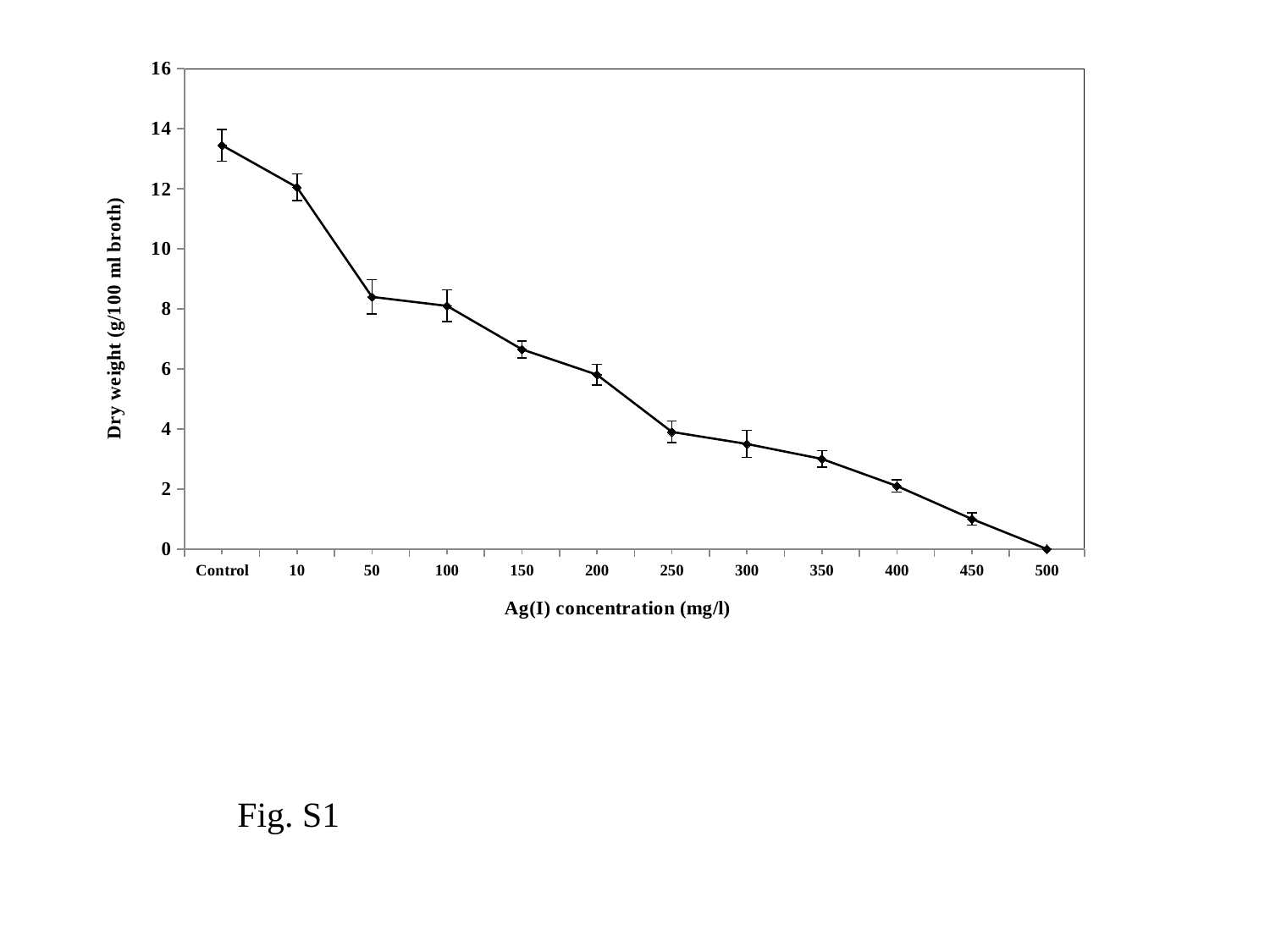

### Chart
| Category | Native biomass |
|---|---|
| Control | 13.450000000000006 |
| 10 | 12.05 |
| 50 | 8.4 |
| 100 | 8.1 |
| 150 | 6.6499999999999995 |
| 200 | 5.8067 |
| 250 | 3.9 |
| 300 | 3.5 |
| 350 | 3.0 |
| 400 | 2.1 |
| 450 | 1.0 |
| 500 | 0.0 |Fig. S1

## Slide 2
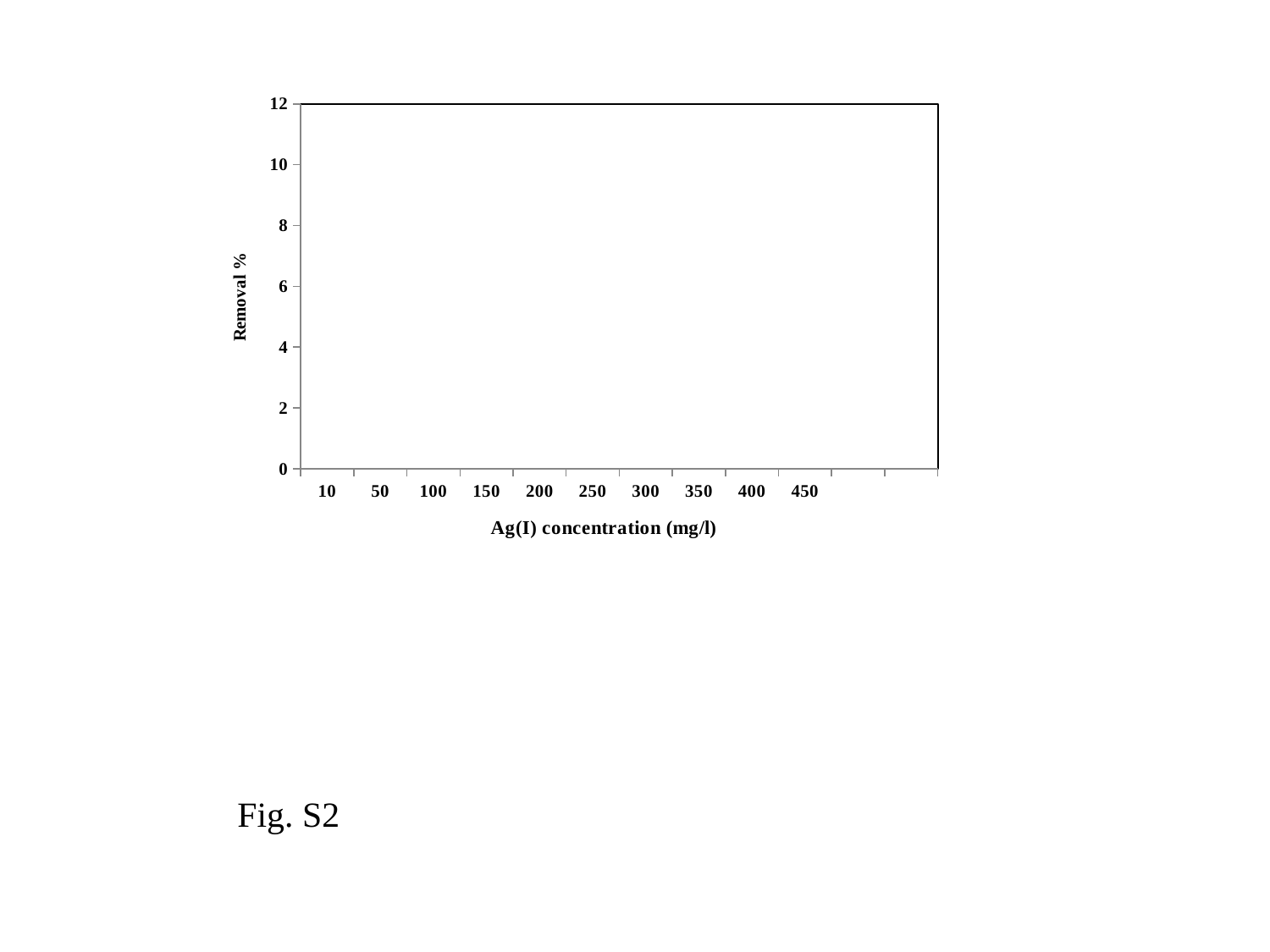

### Chart
| Category | A. awamori |
|---|---|
| 10 | 17.3 |
| 50 | 29.1 |
| 100 | 70.5 |
| 150 | 72.7 |
| 200 | 73.1 |
| 250 | 73.4 |
| 300 | 72.1 |
| 350 | 72.1 |
| 400 | 70.1 |
| 450 | 40.1 |Fig. S2

## Slide 3
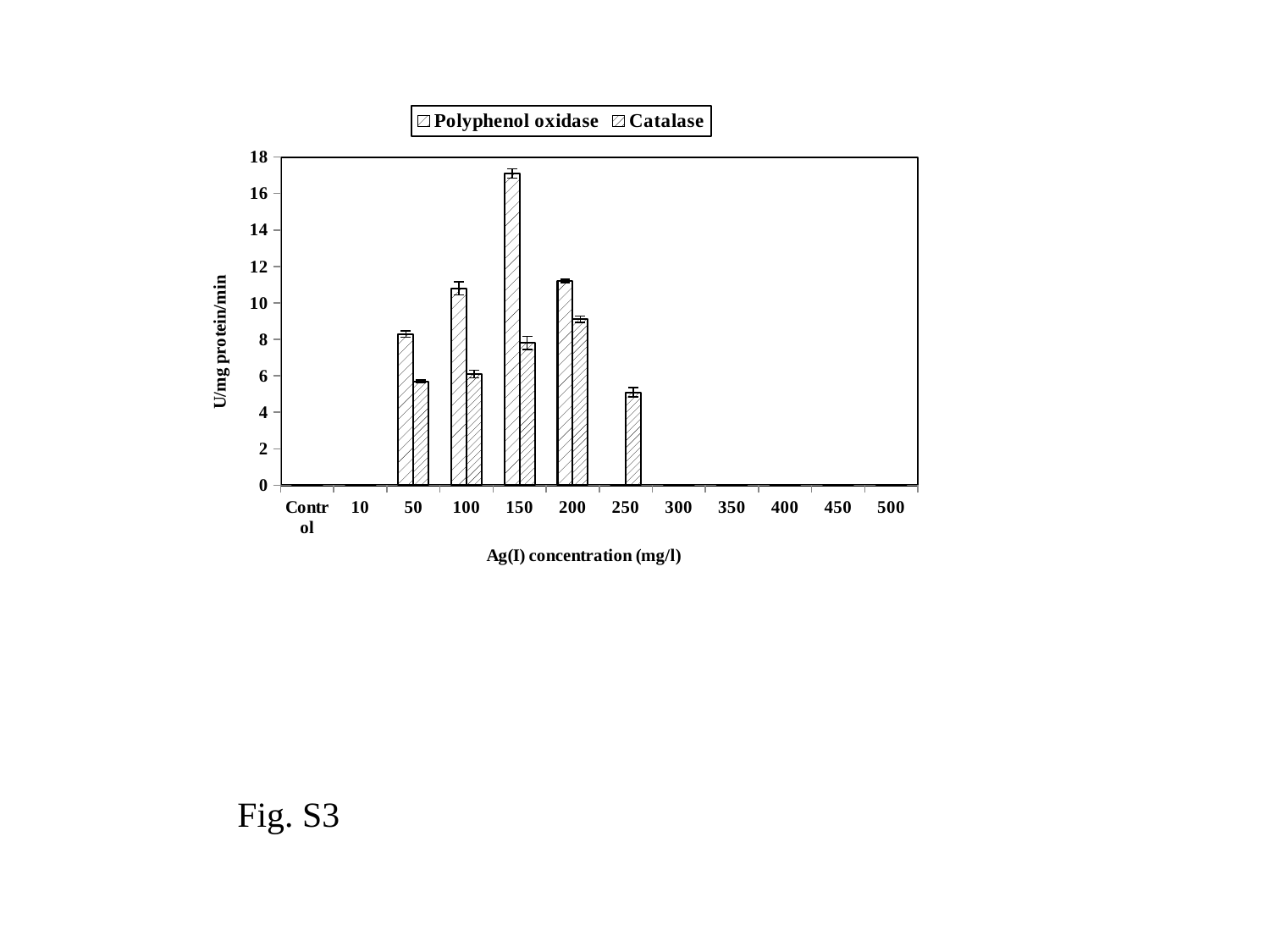

### Chart
| Category | Polyphenol oxidase | Catalase |
|---|---|---|
| Control | 0.0 | 0.0 |
| 10 | 0.016700000000000034 | 0.02000000000000001 |
| 50 | 8.3 | 5.7 |
| 100 | 10.8 | 6.1 |
| 150 | 17.1 | 7.8 |
| 200 | 11.2 | 9.1 |
| 250 | 0.0 | 5.1 |
| 300 | 0.0 | 0.0 |
| 350 | 0.0 | 0.0 |
| 400 | 0.0 | 0.0 |
| 450 | 0.0 | 0.0 |
| 500 | 0.0 | 0.0 |Fig. S3

## Slide 4
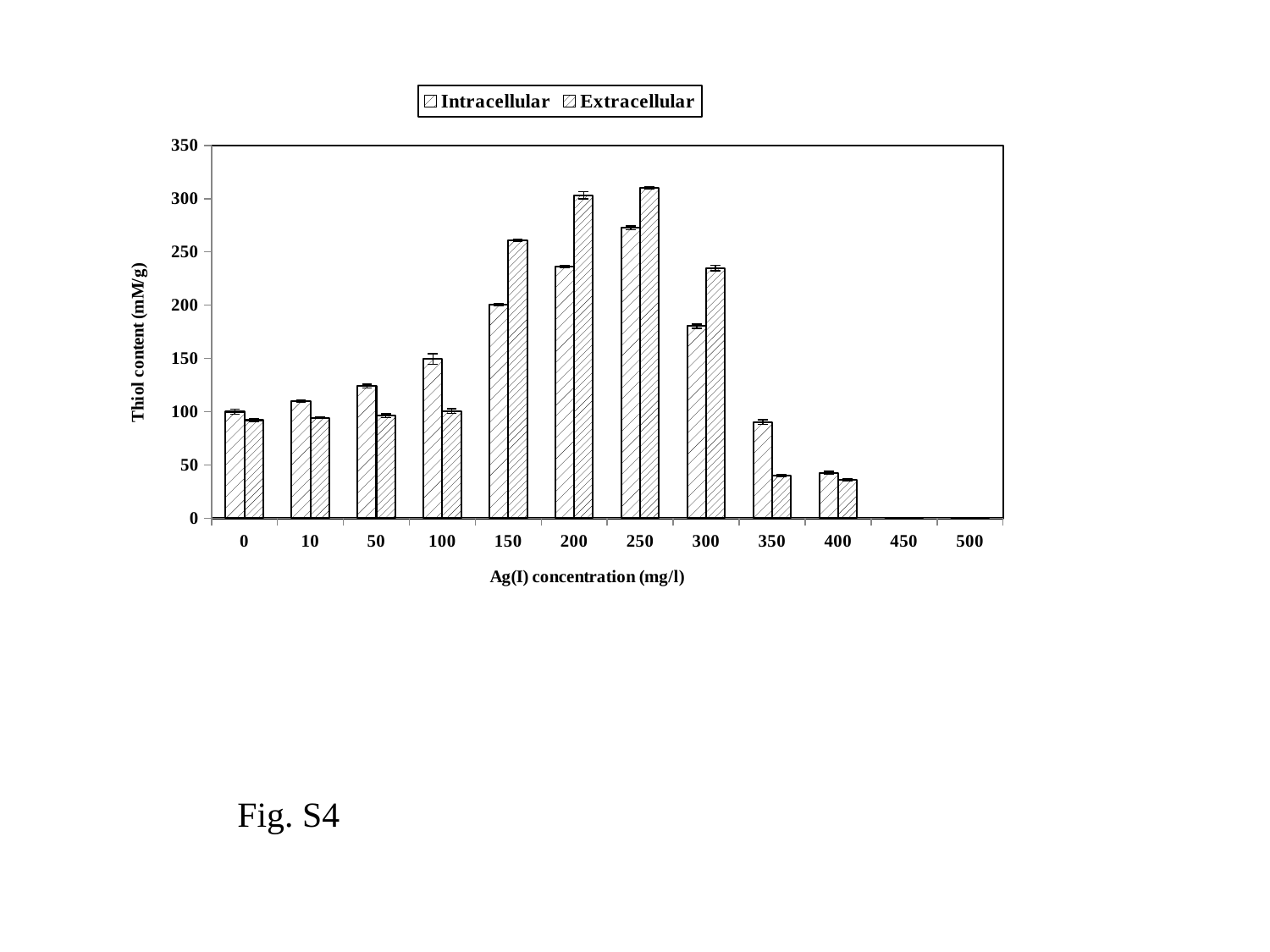

### Chart
| Category | Intracellular | Extracellular |
|---|---|---|
| 0 | 100.0 | 92.1 |
| 10 | 110.1 | 94.3 |
| 50 | 124.0 | 96.2 |
| 100 | 149.55 | 100.5 |
| 150 | 200.4 | 260.7 |
| 200 | 236.2 | 303.1 |
| 250 | 272.5 | 310.0 |
| 300 | 180.3 | 235.0 |
| 350 | 90.3 | 40.0 |
| 400 | 42.7 | 36.0 |
| 450 | 0.0 | 0.0 |
| 500 | 0.0 | 0.0 |Fig. S4

## Slide 5
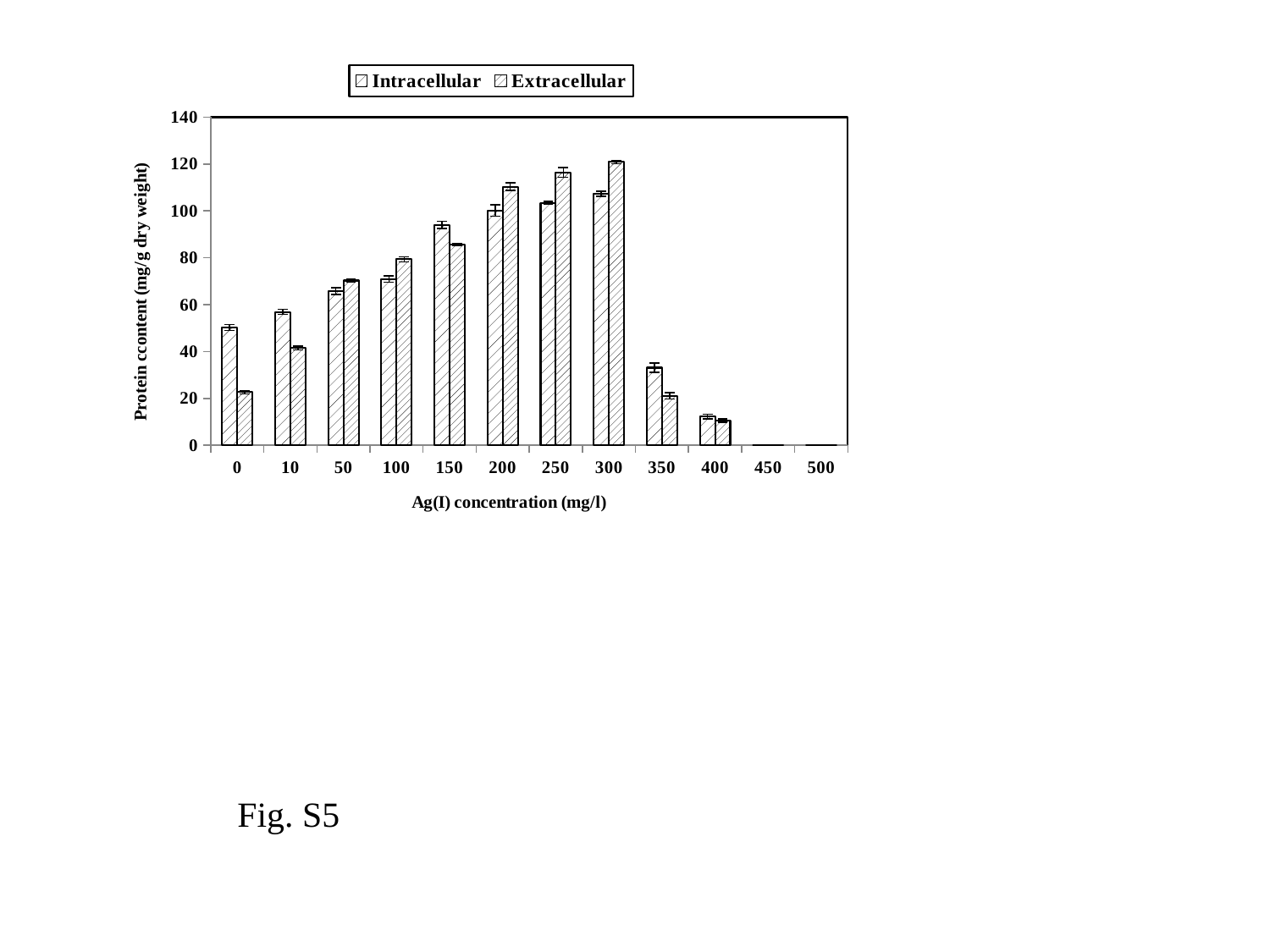

### Chart
| Category | Intracellular | Extracellular |
|---|---|---|
| 0 | 50.3 | 22.7 |
| 10 | 56.9 | 41.5 |
| 50 | 65.8 | 70.3 |
| 100 | 70.9 | 79.4 |
| 150 | 94.1 | 85.5 |
| 200 | 100.2 | 110.3 |
| 250 | 103.4 | 116.45 |
| 300 | 107.3 | 120.8667 |
| 350 | 33.1 | 21.1 |
| 400 | 12.2 | 10.5 |
| 450 | 0.0 | 0.0 |
| 500 | 0.0 | 0.0 |Fig. S5

## Slide 6
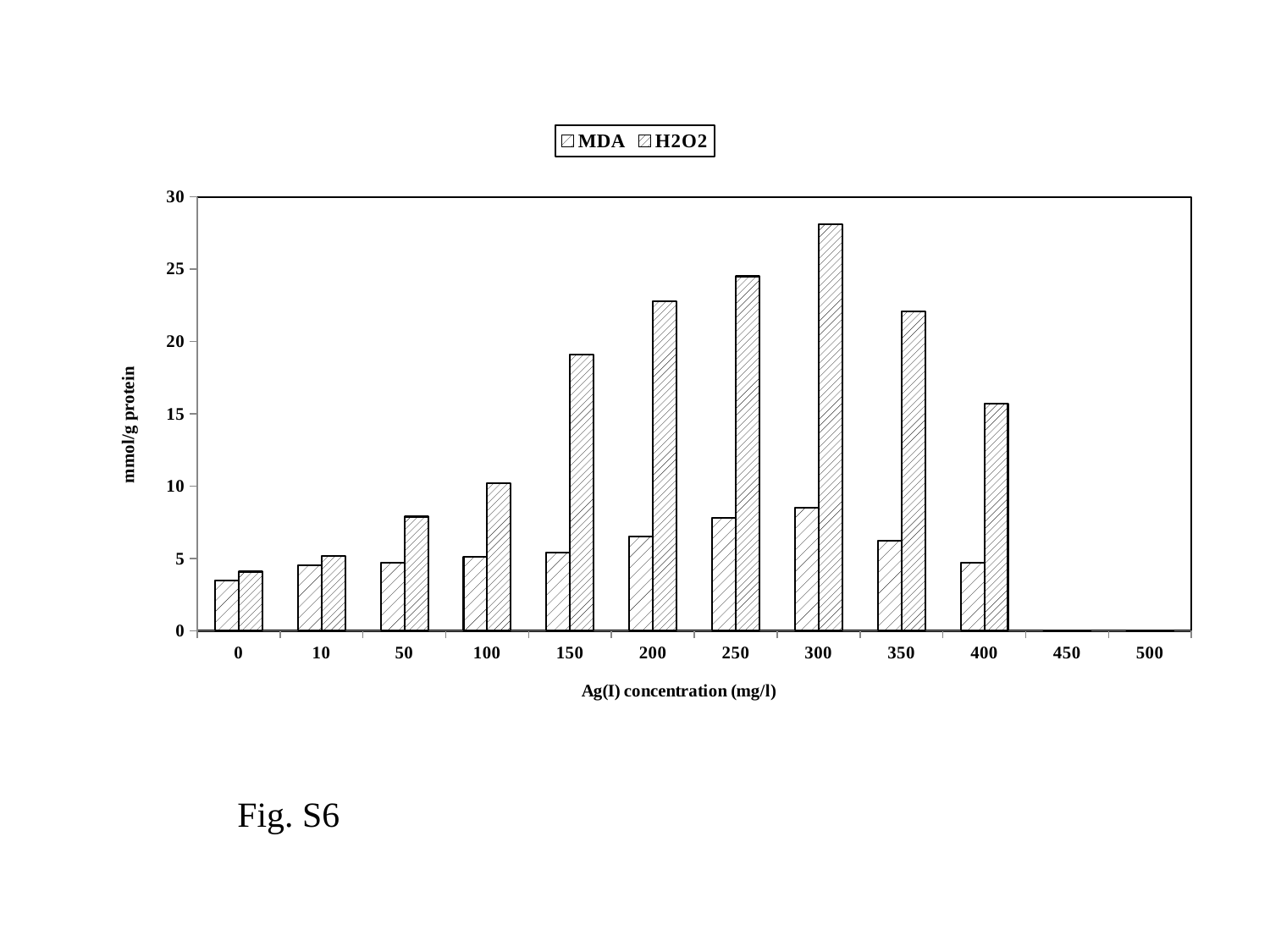

### Chart
| Category | MDA | H2O2 |
|---|---|---|
| 0 | 3.5 | 4.09 |
| 10 | 4.5 | 5.1599999999999975 |
| 50 | 4.7 | 7.89 |
| 100 | 5.1 | 10.200000000000001 |
| 150 | 5.4 | 19.1 |
| 200 | 6.5 | 22.8 |
| 250 | 7.8 | 24.5 |
| 300 | 8.5 | 28.1 |
| 350 | 6.2 | 22.1 |
| 400 | 4.7 | 15.7 |
| 450 | 0.0 | 0.0 |
| 500 | 0.0 | 0.0 |Fig. S6

## Slide 7
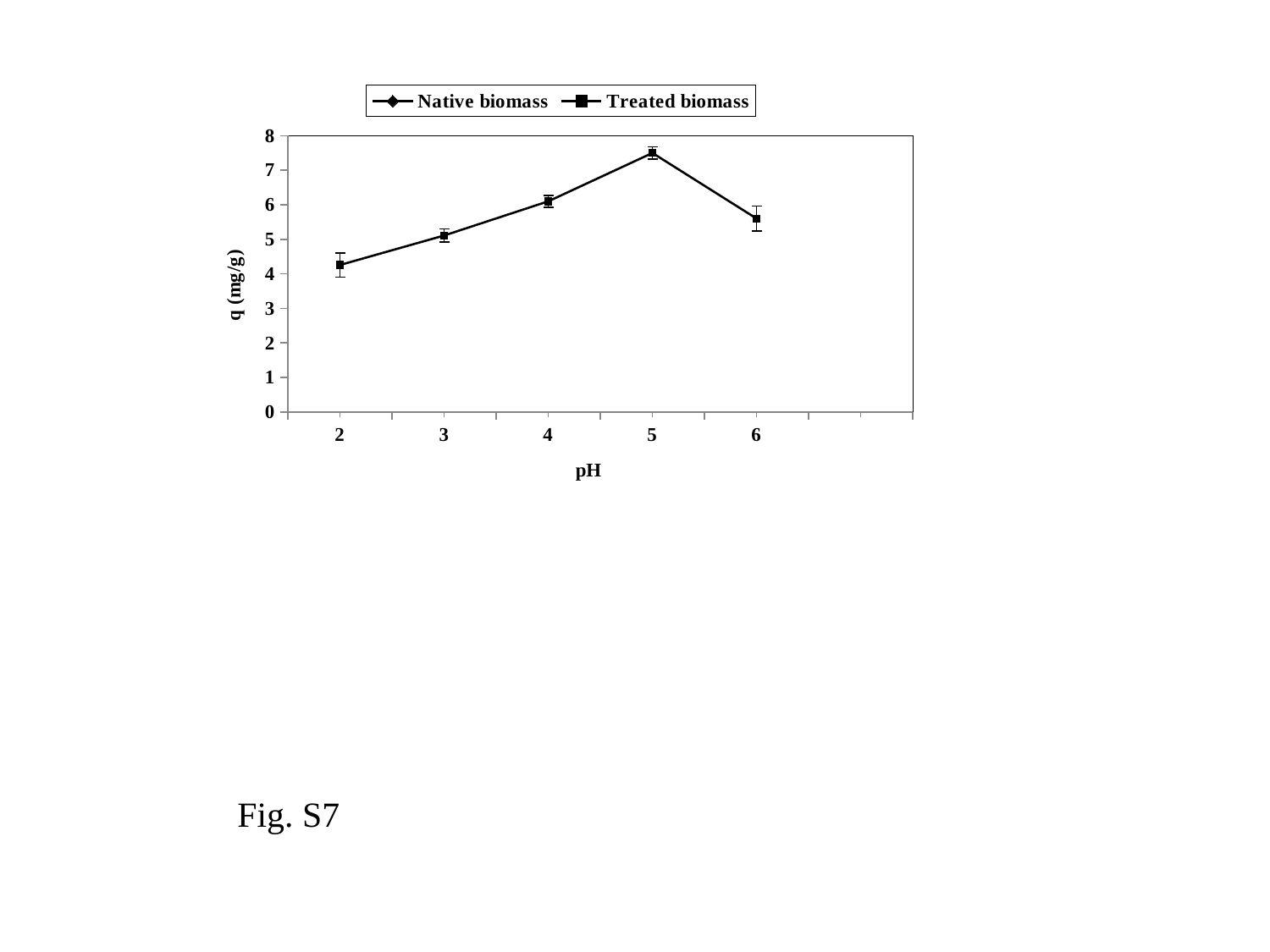

### Chart
| Category | Native biomass | Treated biomass |
|---|---|---|
| 2 | 5.1899999999999995 | 4.2567 |
| 3 | 7.8 | 5.1099999999999985 |
| 4 | 10.200000000000001 | 6.1 |
| 5 | 12.4 | 7.5 |
| 6 | 9.200000000000001 | 5.6 |Fig. S7

## Slide 8
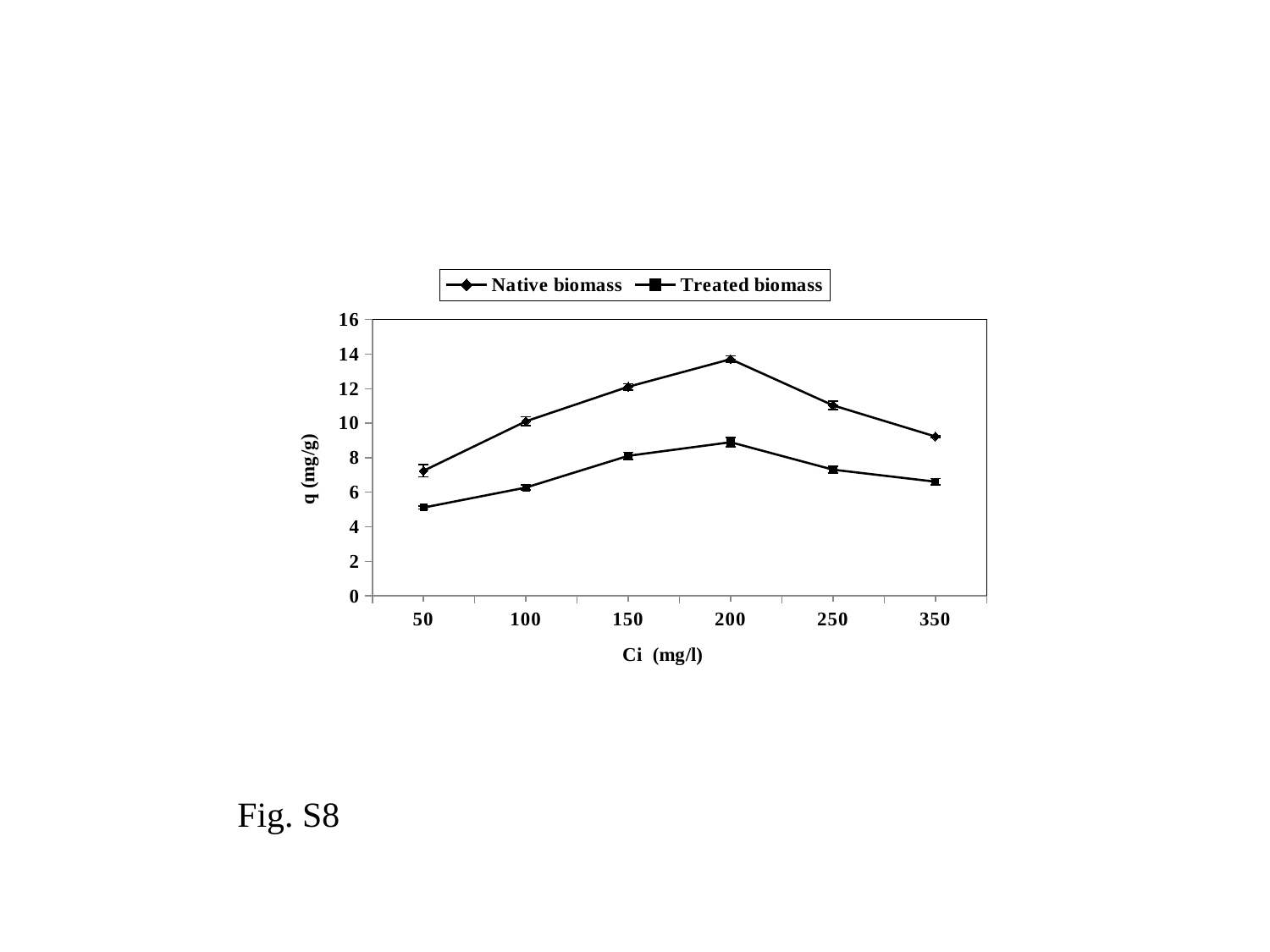

### Chart
| Category | Native biomass | Treated biomass |
|---|---|---|
| 50 | 7.243300000000001 | 5.1099999999999985 |
| 100 | 10.11 | 6.270000000000001 |
| 150 | 12.11 | 8.11 |
| 200 | 13.71 | 8.9 |
| 250 | 11.0333 | 7.31 |
| 350 | 9.23 | 6.6099999999999985 |Fig. S8

## Slide 9
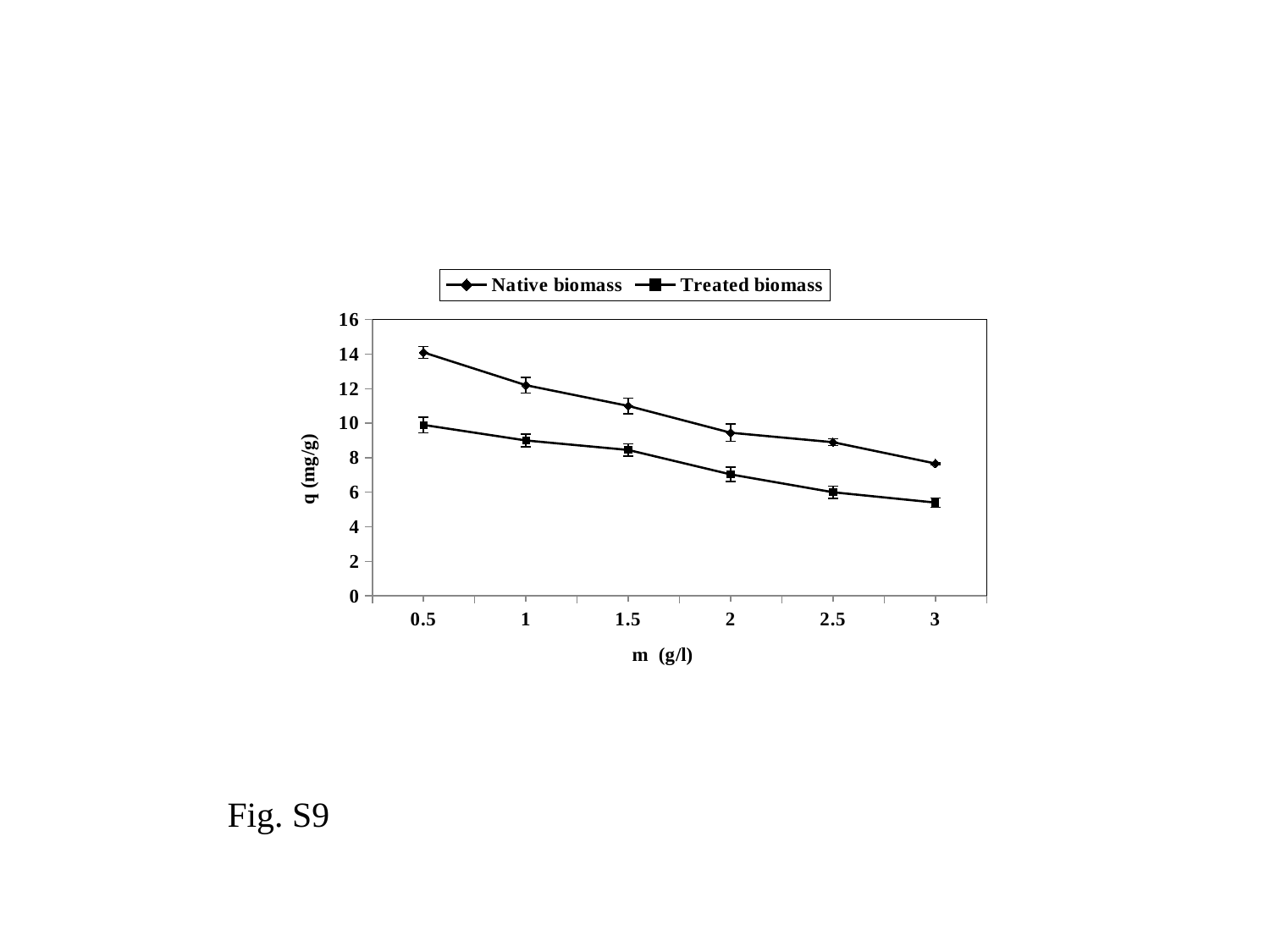

### Chart
| Category | Native biomass | Treated biomass |
|---|---|---|
| 0.5 | 14.1 | 9.9 |
| 1 | 12.2 | 9.0 |
| 1.5 | 11.0 | 8.450000000000003 |
| 2 | 9.450000000000003 | 7.033300000000001 |
| 2.5 | 8.9 | 6.0 |
| 3 | 7.6667 | 5.4 |Fig. S9

## Slide 10
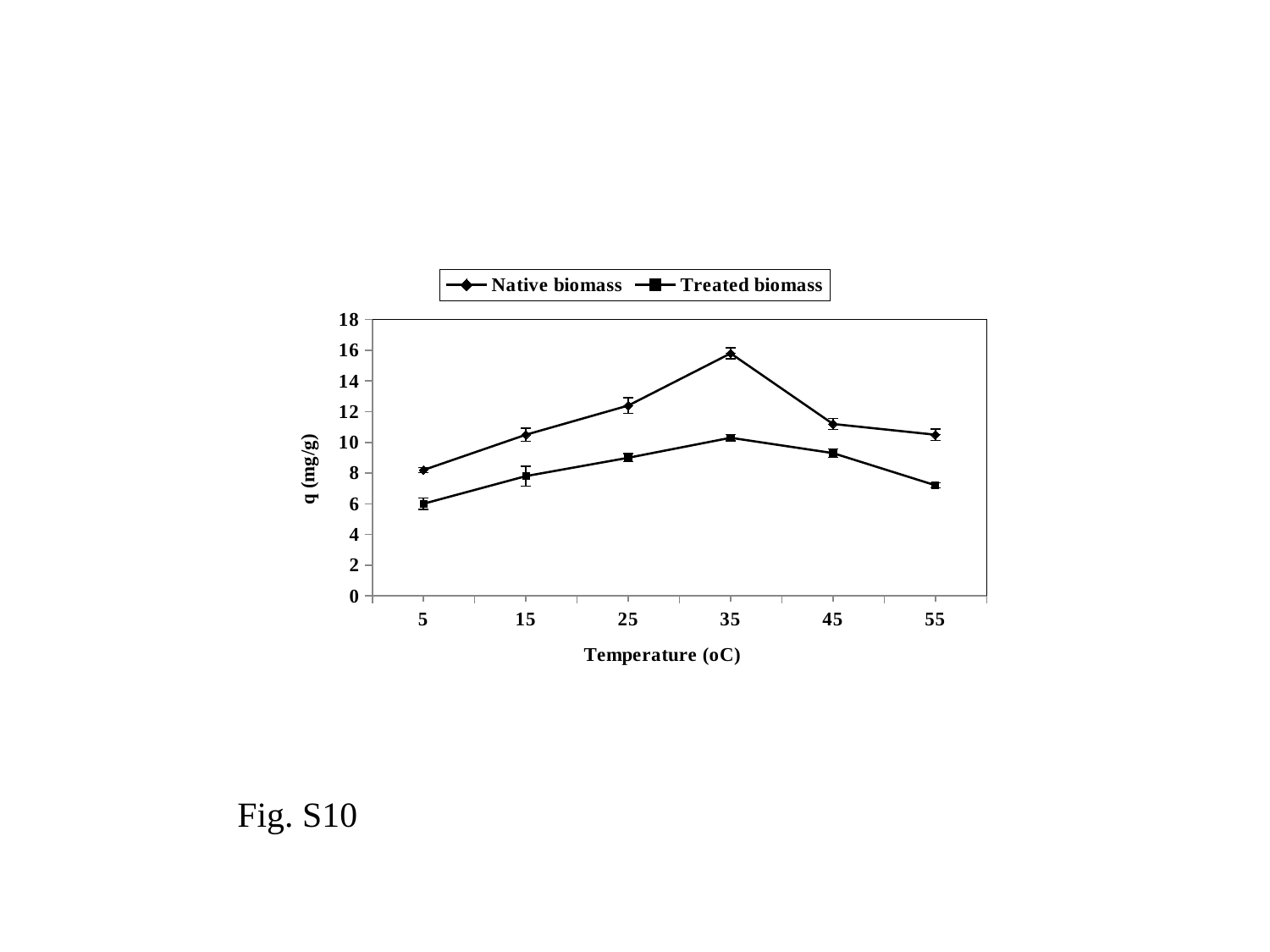

### Chart
| Category | Native biomass | Treated biomass |
|---|---|---|
| 5 | 8.200000000000001 | 6.0 |
| 15 | 10.5 | 7.8 |
| 25 | 12.4 | 9.0 |
| 35 | 15.8 | 10.3 |
| 45 | 11.2 | 9.3 |
| 55 | 10.5 | 7.2 |Fig. S10

## Slide 11
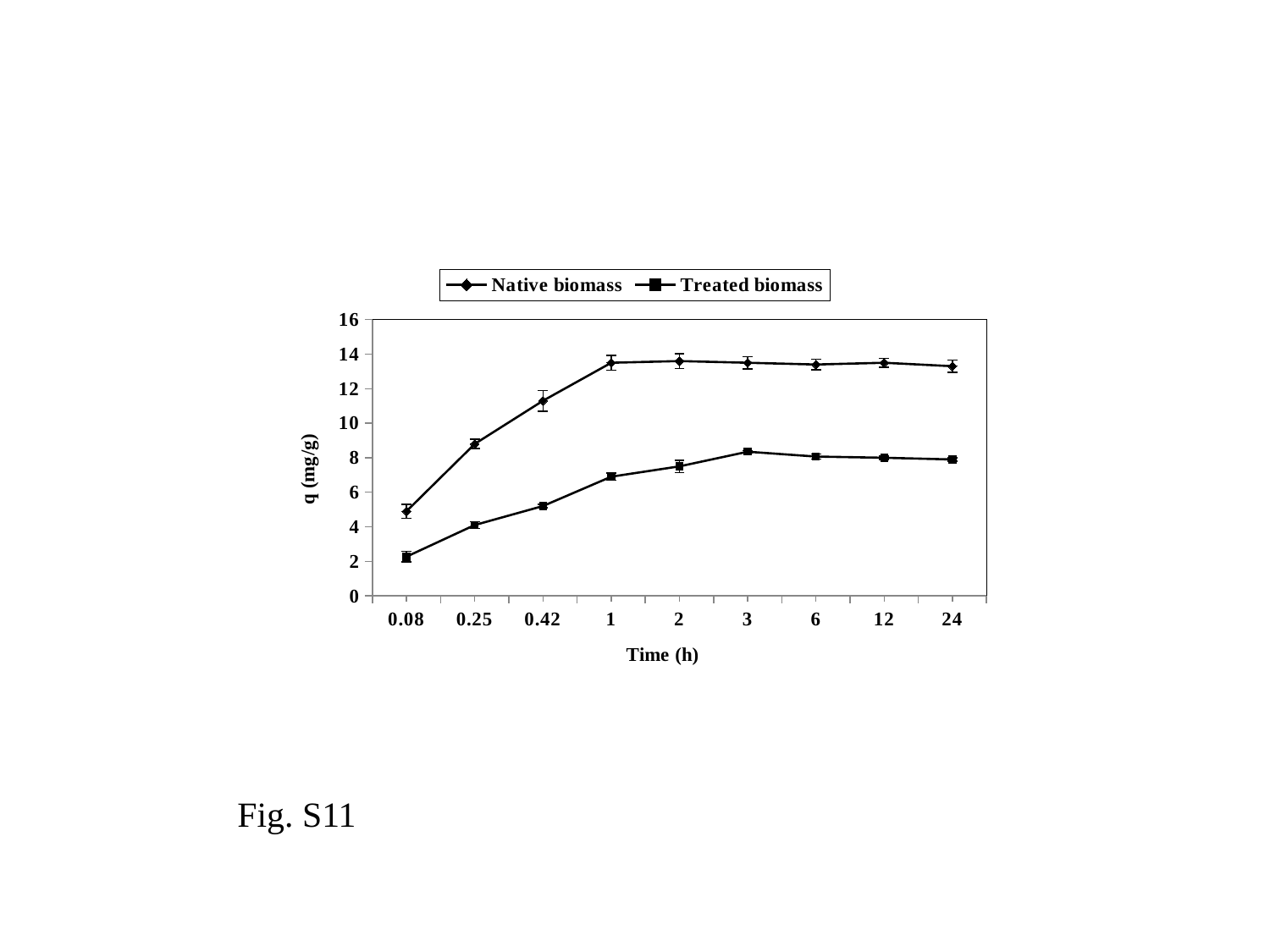

### Chart
| Category | Native biomass | Treated biomass |
|---|---|---|
| 8.0000000000000043E-2 | 4.9 | 2.2667 |
| 0.25 | 8.8 | 4.1 |
| 0.42000000000000032 | 11.3 | 5.2 |
| 1 | 13.5 | 6.9 |
| 2 | 13.6 | 7.5 |
| 3 | 13.5 | 8.350000000000007 |
| 6 | 13.4 | 8.066700000000003 |
| 12 | 13.5 | 8.0 |
| 24 | 13.3 | 7.9 |Fig. S11
